# In silico design of a multiepitope vaccine against antibiotic drug-resistant *Acinetobacter baumannii*

**DOI:** 10.1038/s41598-025-30795-8

**Published:** 2026-04-03

**Authors:** Sudeep Khadka, Muhammad Ajmal Khan, Shahid Iqbal, Amin Ullah, Ajaz Ahmad, Noor Zada Khan, Aishma khattak

**Affiliations:** 1 Department of Biochemistry and Molecular Biology, University of Maryland, Baltimore, MD 21201 USA; 2 Marlene and Stewart Greenebaum Comprehensive Cancer Center, Baltimore, MD 21201 USA; 3 Department of Bioinformatics, Agriculture University Peshawar, Peshawar, Pakistan; 4 Department of Allied Health Sciences, Iqra National University Peshawar, Peshawar, Pakistan; 5 Department of Clinical Pharmacy, College of Pharmacy, King Saud University, 11451 Riyadh, Saudi Arabia; 6 Center of Biotechnology and Microbiology, University of Peshawar, Peshawar, Pakistan; 7 Department of Bioinformatics, Shaheed Benazir Bhutto Women University Peshawar, Peshawar, Pakistan; 8 Center for Stem Cell Biology & Regenerative Medicine, School of medicine University of Maryland Baltimore, Baltimore, USA

**Keywords:** *Acinetobacter baumannii*, In-silico, Vaccine, Multiepitope, Multidrug resistance, Computational biology and bioinformatics, Drug discovery, Microbiology

## Abstract

*Acinetobacter baumannii*, a member of the ESKAPE pathogens, causes approximately 722,000 infections annually and is notorious for rapidly developing resistance to multiple antibiotics, resulting in poor clinical outcomes. Due to the rapid emergence of drug-resistant strains and limited effective treatments, the World Health Organization has listed A. baumannii as a top-priority pathogen for vaccine development, highlighting the urgent need for novel therapeutic strategies. In this study, we employed an in silico approach to design a multiepitope vaccine (MEV) targeting multidrug-resistant strains by analyzing RNA-seq data from meropenem-resistant isolates. The vaccine non-toxicity, probably antigenic, and non-allergenic nature was predicated via ToxinPred, VaxiJen v2.0, and AllerTOP v2.0 tools. CTL and HTL epitope prediction was carried out using NetMHCpan and NetMHCIIpan respectively. PEP-FOLD3.5 server predicted T-cell epitopes 3D structure. Molecular docking of the modeled epitopes into the MHC binding grooves was performed using the HADDOCK 2.4 web server for strong binding interactions. MD simulations were carried out using GROMACS version 2021.4 for the MEV stability and PCA was performed to identify dominant conformational states during the simulation. C-ImmSim server was used for the simulation of immune response. RNA-seq analysis from meropenem resistant *A. baumannii* strains, we identified 1,240 significantly upregulated genes linked to resistance mechanisms. From 156 non-homologous proteins, 12 highly conserved, non-allergenic antigenic proteins were selected to predict 12 CTL and 7 HTL epitopes with strong HLA-binding affinity and immunogenic potential. Molecular docking and dynamics simulations confirmed stable epitope-MHC interactions and structural stability of the 335-amino-acid MEV construct, while immune simulations indicated robust activation of innate and adaptive responses. This in silico study lays a promising foundation for vaccine development against A. baumannii and provides a critical step toward future in vitro and in vivo validation.

**Supplementary Information:**

The online version contains supplementary material available at 10.1038/s41598-025-30795-8.

## Introduction

Hospital-associated infections (HAIs), formerly known as nosocomial infections, pose a serious threat to patient safety worldwide. According to the Centers for Disease Control and Prevention (CDC), these infections are acquired during healthcare delivery and can lead to prolonged hospital stays, increased medical costs, and higher mortality rates^1^. Bacteria are the most common pathogen that causes HAIs, followed by fungi and viruses. The most common Gram-positive bacteria that are linked to HAIs include *Staphylococcus aureus, Streptococcus species, and Enterococcus faecalis* and *Enterococcus faecium,* whereas Gram-negative *bacteria Escherichia coli, Klebsiella pneumoniae, Klebsiella oxytoca, Proteus mirabilis, Enterobacter species, Pseudomonas aeruginosa, Acinetobacter baumannii, and Burkholderia cepacia,* are included in this list. *Acinetobacter baumannii (A. baumannii)*, a short, rod-shaped Gram-negative bacterium is considered a troublesome pathogenic bacterium that survives in hospital settings and is considered a major cause of HAIs due to biofilm-forming capability^2,3^. As a member of the group of multidrug-resistant (MDR) organisms ESKAPE (*Enterococcus faecium*, *Staphylococcus aureus*, *Klebsiella pneumoniae*, *A. baumannii*, *Pseudomonas aeruginosa*, and *Enterobacter* spp.), *A. baumannii* poses a serious threat to healthcare environments and is continuously growing, particularly in intensive care units (ICUs), causing high mortality in patients with chronic lung disease, diabetes, immunocompromised conditions, prolonged hospital stays, hospital ventilators, open wounds, and urinary catheters^4^.

Carbapenems, such as imipenem and meropenem, have traditionally served as the first-line treatment for *A. baumannii* infections due to their broad-spectrum activity. However, the growing emergence of carbapenem-resistant and multidrug-resistant *A. baumannii* strains has significantly limited their effectiveness, making treatment increasingly challenging. There is no consensus available to treat carbapenem-resistant *A. baumannii* (CRAB), and several guidelines support varying suggestions^5,6^. CRAB is included by the World Health Organization (WHO) in the updated Bacterial Priority Pathogen List (BPPL) 2024 due to the high antibiotic resistance^7^. Although there is currently no approved vaccine to prevent *A. baumannii*, experimental animal studies have identified several potential antigens, such as K1 capsular polysaccharide, poly-N-acetyl-β-, autotransporter (Ata), glucosamine (PNAG), biofilm-associated protein (Bap), outer membrane vesicles (OMVs), and outer membrane protein A (OmpA)^8–12^. These antigens’ diversity and lack in circulating strains, however, restrict their potential to combat emerging strains of *A. baumannii*, which are susceptible to immunological pressure-induced mutations that downregulate the target antigens. These factors call for different approaches to this public health emergency, highlighting the necessity of creating potent Immunotherapeutics to fight this infection.

Recent advancements in computational biology and bioinformatics have made it possible to rapidly develop effective vaccine constructs, minimizing the need for conventional laboratory techniques^13^. Conventional vaccine development requires considerable time and effort and is primarily based on empirical knowledge; however, as immunoinformatic techniques gain popularity, in silico vaccine development has emerged as an attractive new strategy. Vaccines against a variety of pathogens, including bacteria, viruses, parasites, and tumors, have been developed thus far; the majority of these are related to COVID-19 ^14^. In this study, we employed an in silico approach to design a multi-epitope vaccine (MEV) targeting *A. baumannii*, focusing on upregulated genes associated with antibiotic resistance. Molecular docking and molecular dynamics (MD) simulations demonstrated that the MEV exhibits favorable binding affinity and structural stability, supporting its potential as a vaccine candidate against *A. baumannii*.

## Results

### Transcriptomic profiling reveals meropenem-induced gene expression changes in *Acinetobacter baumannii*

**Fig. 1:**
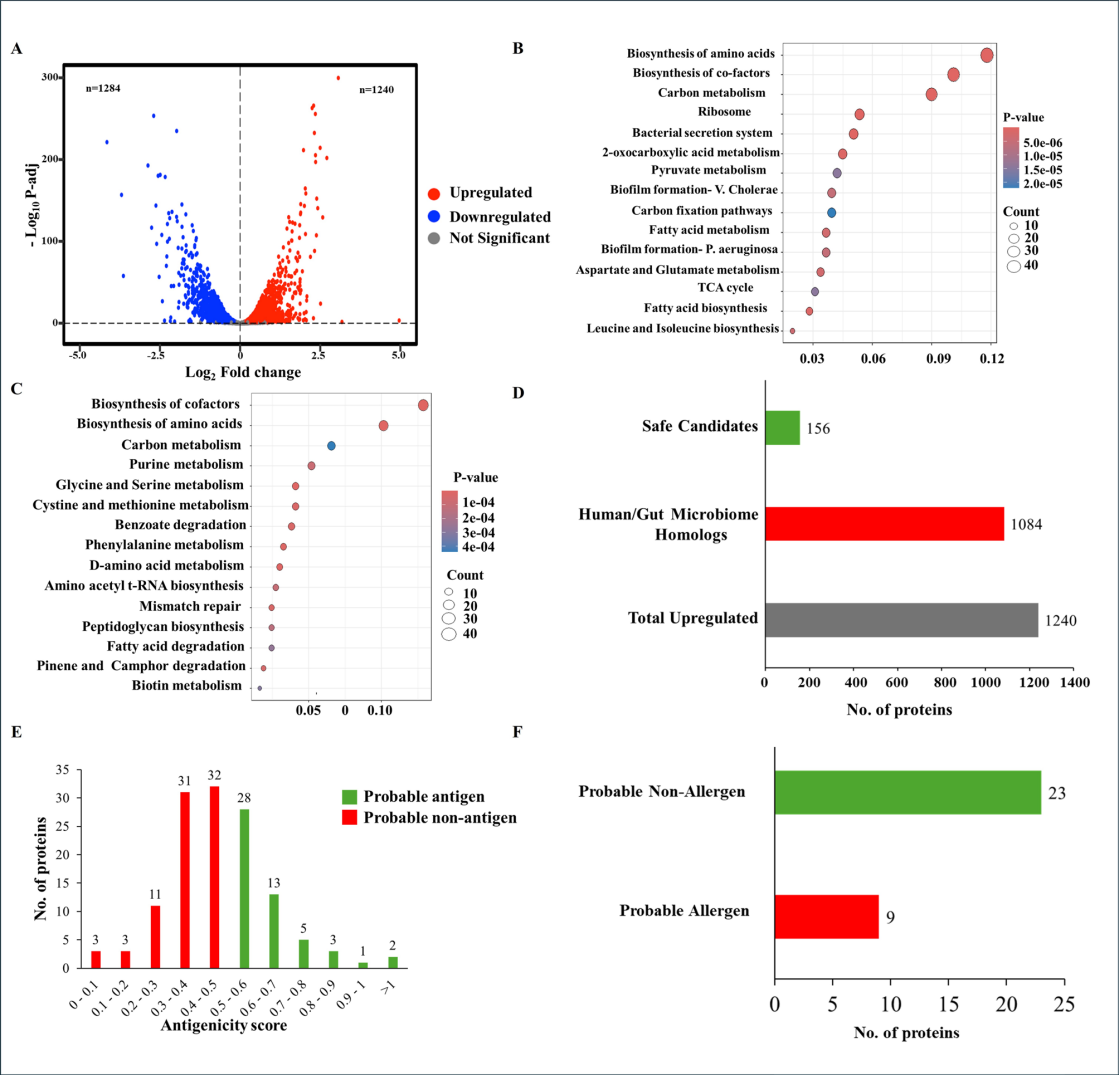
Meropenem-induced gene expression changes in *Acinetobacter baumannii* and prioritization of upregulated antigens for vaccine development. (**a**) Differential gene expression analysis. Volcano plot illustrating global transcriptional changes after 9-h meropenem treatment. Genes significantly upregulated (1,240 genes; shown in red) and significantly downregulated (1,284 genes; shown in blue) were determined using an adjusted p-value < 0.05. (**b**) GO enrichment of upregulated genes. Gene Ontology (GO) enrichment analysis of upregulated genes reveals significant activation of pathways involved in carbon metabolism, bacterial secretion systems, and other metabolic processes. (**c**) GO enrichment of downregulated genes. Downregulated genes show significant enrichment for pathways associated with amino acid biosynthesis and metabolism, indicating suppression of key metabolic functions following antibiotic exposure. (**d**) Identification of non-homologous proteins. BLASTp analysis of the upregulated gene set was performed to exclude proteins with homology to human or common human gut microbiota. A total of 156 proteins were identified as non-homologous and were selected for further immunogenicity screening. (**e**) Antigenicity prediction. Antigenicity analysis of the 156 non-homologous proteins identified 32 proteins with an antigenicity score > 0.5, classifying them as probable antigens. (**f**) Allergenicity filtering and final candidate selection. Among the probable antigens with ≥ 1.5-fold upregulation, allergenicity prediction identified 23 non-allergenic proteins. These were selected as the final set of candidate proteins for potential vaccine development.

We analyzed RNA-seq data following a 9-h treatment of *A. baumannii* cultures with or without meropenem, a carbapenem antibiotic. Differential expression analysis revealed that 1,240 genes were significantly upregulated, and 1,284 genes were significantly downregulated (adjusted p < 0.05) upon meropenem treatment (Fig. 1a). Pathway enrichment analysis identified several biological pathways altered in response to meropenem exposure. Notably, carbon metabolism and the bacterial secretion system were among the top upregulated pathways potentially contributing to antibiotic resistance (Fig. 1b, Fig. S1a). Conversely, multiple amino acid metabolism pathways were significantly downregulated following treatment (Fig. 1c, Fig. S1b). Additionally, genes associated with efflux pumps, particularly those related to outer membrane proteins (OMPs) and the resistance-nodulation-cell division (RND) family, were upregulated (Fig. S1c). This suggests that *A. baumannii* may evade meropenem through active drug efflux mechanisms.

### Selection of upregulated, non-homologous antigenic proteins for vaccine potential

To identify potential vaccine candidates, we focused on genes that were significantly upregulated upon meropenem treatment, as these may contribute to resistance and represent promising targets for intervention. Targeting such genes may not only help overcome resistance but also improve the likelihood of immune recognition due to increased peptide presentation during infection. Protein sequences encoded by the 1,240 upregulated genes were extracted and screened for host similarity using BLAST searches against the human genome and common human gut microbiota. To minimize off-target effects and cross-reactivity, only non-homologous proteins were retained. The filtering resulted in 156 proteins deemed safe for further analysis, while 1,084 homologous proteins were excluded (Fig. 1d). Next, we assessed the antigenic potential of the 156 non-homologous proteins. Antigenicity analysis identified 52 proteins as probable antigens (Fig. 1e). From these, 32 proteins were selected based on their expression level, specifically those upregulated by at least 1.5-fold. Allergenicity screening further narrowed the candidates, identifying 23 proteins as non-allergenic and suitable for downstream vaccine development (Fig. 1f).

### Conservation analysis of protein candidates across virulent *A. baumannii* strains

**Fig. 2:**
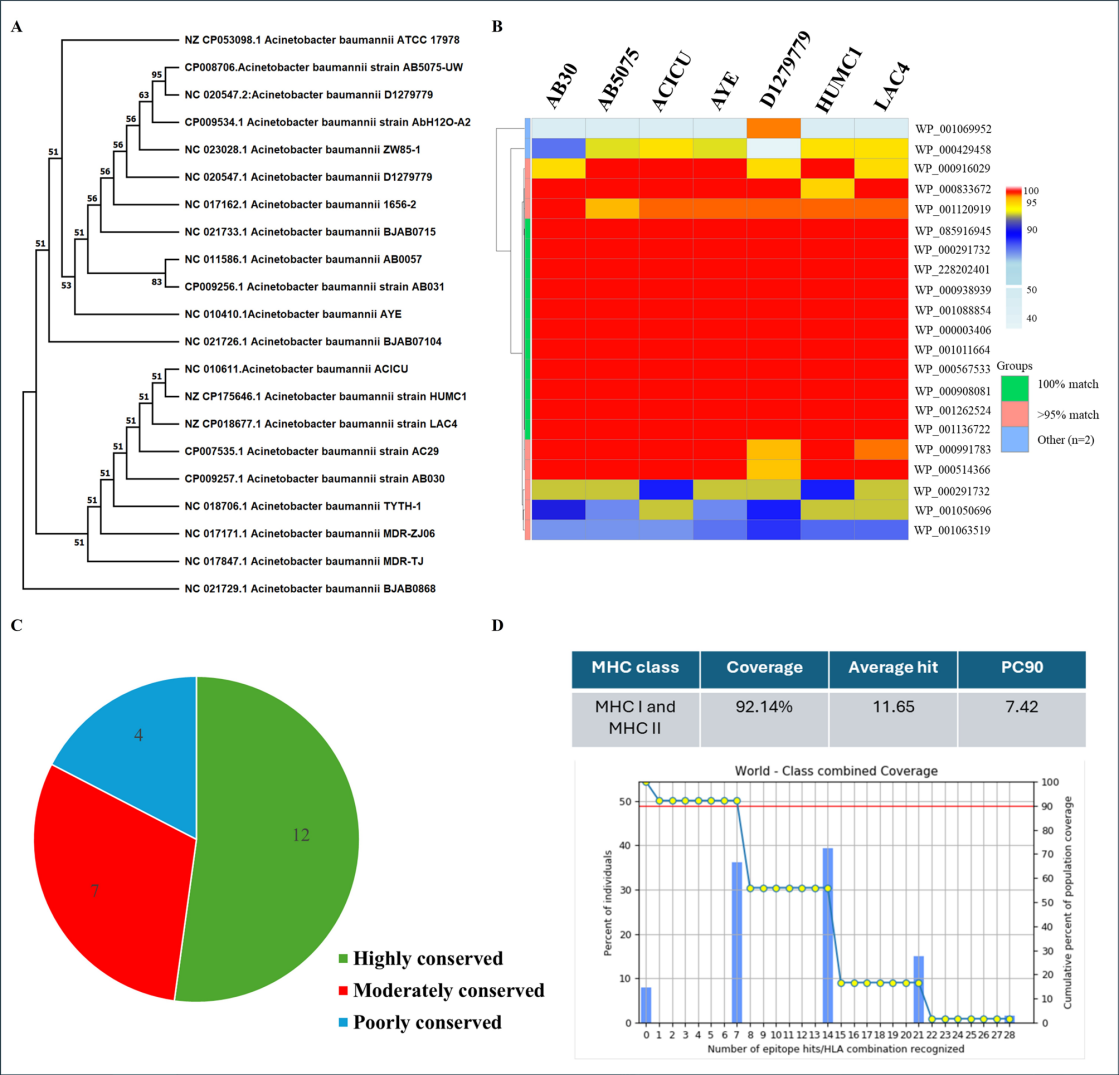
Conservation of candidate vaccine antigens across virulent *Acinetobacter baumannii* strains. (**a**) Phylogenetic analysis of *A. baumannii* strains. Maximum likelihood phylogeny of 21 *A. baumannii* strains constructed using MEGA X. Codon positions included 1st, 2nd, 3rd, and noncoding sites, resulting in a final dataset of 1,545 aligned positions. The tree topology reflects the genetic diversity and evolutionary relationships among the strains. (**b**) Conservation of candidate proteins across virulent strains. Heatmap showing BLASTp sequence identity of the 23 final candidate proteins across seven virulent *A. baumannii* strains (AB30, AB5075, ACICU, AYE, D1279779, HUMC1, and LAC4). Twelve proteins showed complete conservation (100% identity), seven were moderately conserved (> 95% identity), and four displayed lower conservation (< 95% identity). (**c**) Distribution of protein conservation levels. Pie chart summarizing the conservation categories defined in panel (b). Of the 23 candidate proteins, 12 were classified as highly conserved (100% identity), highlighting their potential suitability for broad-spectrum vaccine design. (**d**) Predicted global population coverage of CTL and HTL epitopes. Cytotoxic T lymphocyte (CTL) and helper T lymphocyte (HTL) epitopes derived from the conserved proteins were analyzed using the IEDB Population Coverage tool. The table and graph display the predicted global HLA allele coverage (%) for the selected epitope set. “Average” denotes the mean number of epitopes-HLA combinations recognized per individual, while “pc90” indicates the minimum number of epitopes-HLA hits expected to be recognized in 90% of the population.

We performed phylogenetic analysis among different strains of *A. baumannii* to confirm the diversity through MEGA X. Our analysis showed clustering of the strains in distinct clades, demonstrating the isolates’ significant genomic diversity and indicating potential variations in resistance profiles, pathogenicity, or origin (Fig. 2a). We next performed BLASTp analysis on the 23 protein candidates to assess their conservation among the most virulent strains of *A. baumannii*, specifically AB30, AB5075, ACICU, AYE, D1279779, HUMC1, and LAC4. Twelve proteins were found to be 100% conserved across these strains, seven were moderately conserved (showing > 95% sequence homology), and four were poorly conserved (< 95%) (Fig. 2b, 2c). The 12 fully conserved proteins were selected as potential candidates for further downstream analysis and are listed in Table 1 (Fig. S2a). Furthermore, we confirmed the sequence similarity through phylogenetic analysis and found the proteins clustered in the same clade confirmed by the tree visualized through iTOL (Fig. S2b).

**Table 1:** Summary of candidate proteins for MEV construction.

Protein	Protein name	Antigen probability	Status	Allergy-status	Conserved
WP_000003406	Sulfurtransferase TusA	0.6473	Probable antigen	Probable non-allergen	High
WP_000567533	HPr family phosphocarrier protein	0.7446	Probable antigen	Probable non-allergen	High
WP_000833672	succinate dehydrogenase	0.6795	Probable antigen	Probable non-allergen	High
WP_000908081	Sec-independent protein translocase subunit TatA	0.5241	Probable antigen	Probable non-allergen	High
WP_000938939	Hypothetical protein	0.5744	Probable antigen	Probable non-allergen	High
WP_000973732	Arginine N-succinyltransferase	0.5933	Probable antigen	Probable non-allergen	High
WP_001011664	MotA/TolQ/ExbB proton channel family protein	0.5501	Probable antigen	Probable non-allergen	High
WP_001088854	DUF4870 family protein	0.8016	Probable antigen	Probable non-allergen	High
WP_001136722	30S ribosomal protein S21	0.5069	Probable antigen	Probable non-allergen	High
WP_001262524	RNA polymerase-binding protein DksA	0.6229	Probable antigen	Probable non-allergen	High
WP_085916945	cyd operon YbgE family protein	1.2296	Probable antigen	Probable non-allergen	High
WP_228202401	H-NS family nucleoid-associated regulatory protein	0.5638	Probable antigen	Probable non-allergen	High

### Cytotoxic T lymphocyte and helper T lymphocyte epitopes prediction

Cytotoxic T lymphocyte (CTL) epitope prediction was conducted on 12 protein candidates that were fully conserved across virulent *A. baumannii* strains. Using the NetMHCpan 4.1 server, 9-mer peptides were evaluated against a representative panel of human HLA class I alleles, including multiple HLA-A and HLA-B subtypes. Predicted epitopes were subsequently assessed for immunogenicity using the IEDB Immunogenicity tool and screened for toxicity via ToxinPred. Epitopes demonstrating high immunogenicity, non-toxicity, and strong HLA-binding affinity were selected. This process yielded 12 high-confidence CTL epitopes, as summarized in Table 2. Similarly, helper T lymphocyte (HTL) epitope prediction was performed on the same 12 conserved protein candidates. Fifteen-mer peptides were analyzed using the NetMHCIIpan 4.0 server against a representative panel of human HLA class II alleles, including HLA-DRB1, DRB3, DRB4, and DRB5 subtypes. Based on their immunogenicity, safety profile, and binding strength, 7 high-confidence HTL epitopes were selected for vaccine construction and are listed in Table 3. Together, the selected CTL and HTL epitopes span 10 of the 12 conserved proteins, ensuring broad antigenic coverage.

**Table 2:** Cytotoxic T lymphocyte (CTL) target peptides with physicochemical profiles.

Peptide	ID	Immunogenicity score	Toxicity Prediction	SVM Score	Hydrophobicity	Steric hindrance	Sidebulk	Hydropathicity	Amphipathicity	Hydrophilicity	Net Hydrogen	Charge	pI	Mol wt
AAAWIWIVY	WP_001088854	0.71014	Non-Toxin	-0.44	0.39	0.6	0.6	1.72	0	-1.74	0.33	0	5.88	1092.43
YLAWNIIAF	WP_001088854	0.48975	Non-Toxin	-1.27	0.32	0.63	0.63	1.5	0	-1.6	0.44	0	5.88	1110.45
QEEEFAIEL	WP_001262524	0.4549	Non-Toxin	-0.71	-0.12	0.65	0.65	-0.51	0.7	0.62	0.67	-4	3.59	1107.31
LSIWHVVIF	WP_000908081	0.44905	Non-Toxin	-1.24	0.38	0.56	0.56	2.12	0.16	-1.61	0.33	0.5	7.1	1113.51
FLPFIFLFF	WP_001088854	0.41304	Non-Toxin	-1.03	0.53	0.62	0.62	2.72	0	-1.99	0	0	5.88	1190.62
YAIWGIQIF	WP_000833672	0.40437	Non-Toxin	-1.26	0.32	0.65	0.65	1.33	0.14	-1.54	0.44	0	5.88	1110.46
SRDWFIQRV	WP_000833672	0.40185	Non-Toxin	-1.03	-0.33	0.66	0.66	-0.69	0.68	0.03	1.44	1	9.95	1206.49
TVWGIFHAL	WP_001011664	0.39596	Non-Toxin	-1.05	0.29	0.54	0.54	1.32	0.16	-1.38	0.33	0.5	7.1	1043.37
QIIEIAENV	WP_228202401	0.3911	Non-Toxin	-0.71	0.05	0.68	0.68	0.61	0.42	-0.11	0.67	-2	3.8	1028.31
MSIATWTVF	WP_001011664	0.38576	Non-Toxin	-1.06	0.24	0.61	0.61	1.34	0	-1.28	0.44	0	5.88	1055.38
AVIIAVFVY	WP_000833672	0.377	Non-Toxin	-1.11	0.47	0.66	0.66	2.97	0	-1.54	0.11	0	5.88	994.37
TFSIEIVTL	WP_000291732	0.36052	Non-Toxin	-1.33	0.21	0.62	0.62	1.57	0.14	-0.77	0.44	-1	4	1022.34

**Table 3:** Helper T lymphocyte (HTL) target peptides with physicochemical profiles.

Peptide	ID	Immunogenicity score	Prediction	SVM Score	Hydrophobicity	Steric hindrance	Sidebulk	Hydropathicity	Amphipathicity	Hydrophilicity	Net Hydrogen	Charge	pI	Mol wt
RKREFYEKPTQERKR	WP_001136722	99.5419	Non-Toxin	-1.4	-0.83	0.65	0.65	-2.97	1.72	1.67	1.93	4	10.43	2051.54
TLRLVIDGTDEEQAL	WP_000567533	98.7825	Non-Toxin	-0.81	-0.15	0.63	0.63	-0.13	0.42	0.35	0.8	-3	3.92	1673.08
EPRYRNKNNAEETWT	WP_228202401	98.3272	Non-Toxin	-0.33	-0.55	0.63	0.63	-2.49	0.83	0.77	1.53	0	6.57	1908.22
ADKGISVLGTIASIA	WP_001011664	98.0262	Non-Toxin	-0.91	0.12	0.62	0.62	1.09	0.24	-0.27	0.4	0	6.19	1415.87
AGVLADVRKREFYEK	WP_001136722	97.8176	Non-Toxin	-1	-0.32	0.66	0.66	-0.69	0.99	0.69	1.07	1	8.83	1781.25
GDVVEVLATDPSTSW	WP_000003406	96.9282	Non-Toxin	-1.14	-0.01	0.6	0.6	0.12	0.08	-0.09	0.53	-3	3.5	1575.9
HTEVVVEGDKASAKR	WP_001262524	96.2953	Non-Toxin	-0.62	-0.3	0.6	0.6	-0.78	0.92	0.79	0.93	0.5	7.11	1626.01

To evaluate the global applicability of the designed vaccine, population coverage analysis was performed using the IEDB Population Coverage tool based on the selected HLA class I and II alleles. The combined CTL and HTL epitopes exhibited an estimated 92.14% projected global population coverage, with an average of 11.65 epitope-HLA hits per individual and a pc90 value of 7.42, indicating that 90% of the world population is expected to recognize at least seven epitope-HLA combinations (Fig. 2d, Table 4). These results suggest that the designed multiepitope vaccine construct provides extensive worldwide HLA representation and potential immunogenic responsiveness across diverse ethnic populations

**Table 4:**
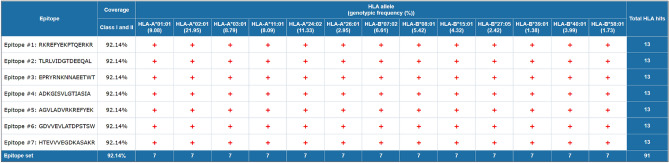
Global HLA population coverage of selected CTL and HTL epitopes.

### Molecular docking of predicted T-cell epitopes with MHC class I and II molecules

**Fig. 3:**
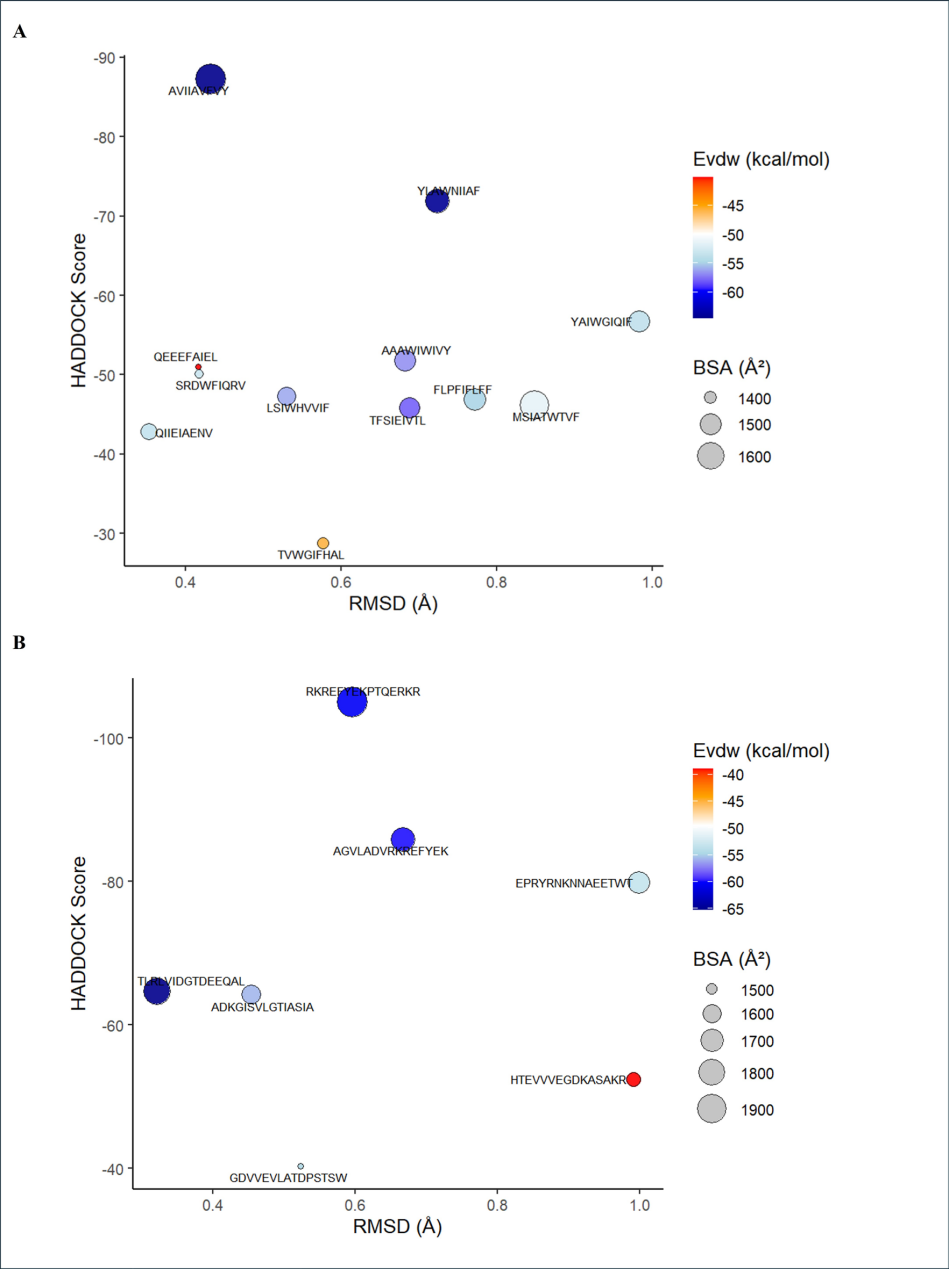
Molecular docking analysis of predicted T-cell epitopes with MHC molecules. (**a**) Docking performance of CTL epitopes. Dot plot displaying the molecular docking scores of cytotoxic T lymphocyte (CTL) epitopes docked with their corresponding MHC class I alleles. All CTL peptides showed favorable binding energies, supported by low van der Waals interaction energies, indicating strong and stable peptide–MHC class I interactions. (**b**) Docking performance of HTL epitopes. Dot plot showing the docking scores of helper T lymphocyte (HTL) epitopes with their respective MHC class II molecules. Similar to the CTL set, all HTL peptides demonstrated favorable docking energies and stable interactions as reflected by favorable van der Waals contributions.

All predicted T-cell epitopes were successfully modeled into stable 3D structures using the PEP-FOLD 3.5 server and docked into their respective MHC molecules. HTL and CTL peptides demonstrated acceptable and stable interactions with their respective MHC molecules (CTL epitopes with MHC class I and HTL epitopes with MHC class II), supported by favorable HADDOCK scores, low RMSD values, substantial van der Waals energies (EVDW), and buried surface areas (BSA). HADDOCK scores for HTL peptides ranged from -105.06 to -40.32, while those for CTL peptides ranged from -87.29 to -28.79, indicating energetically favorable binding across all peptides (Fig. 3a, b). RMSD values were consistently low for both groups, spanning 0.322 to 0.999 Å for HTL peptides and 0.353 to 0.983 Å for CTL peptides, reflecting stable docking conformations (Fig. 3a, b). Importantly, the EVDW values ranged from -65.24 to -39.01 kcal/mol for HTL peptides and from -64.54 to -40.18 kcal/mol for CTL peptides, indicating meaningful non-covalent van der Waals interactions (Fig. 3a, b). Likewise, BSA values, indicative of the extent of peptide–protein interface contact, ranged from 1485 to 1937 Å^2^ for HTL peptides and from 1381 to 1671 Å^2^ for CTL peptides, further supporting stable peptide–MHC interactions (Fig. 3a, b). A detailed summary of the docking interactions is presented in Supplementary Figures S3a and S3b. Together, the docking data confirm that all peptides establish viable and stable interactions with their respective MHC molecules, underscoring their potential as promising candidates for subsequent vaccine assembly.

### Vaccine construct design and structural evaluation

**Fig. 4:**
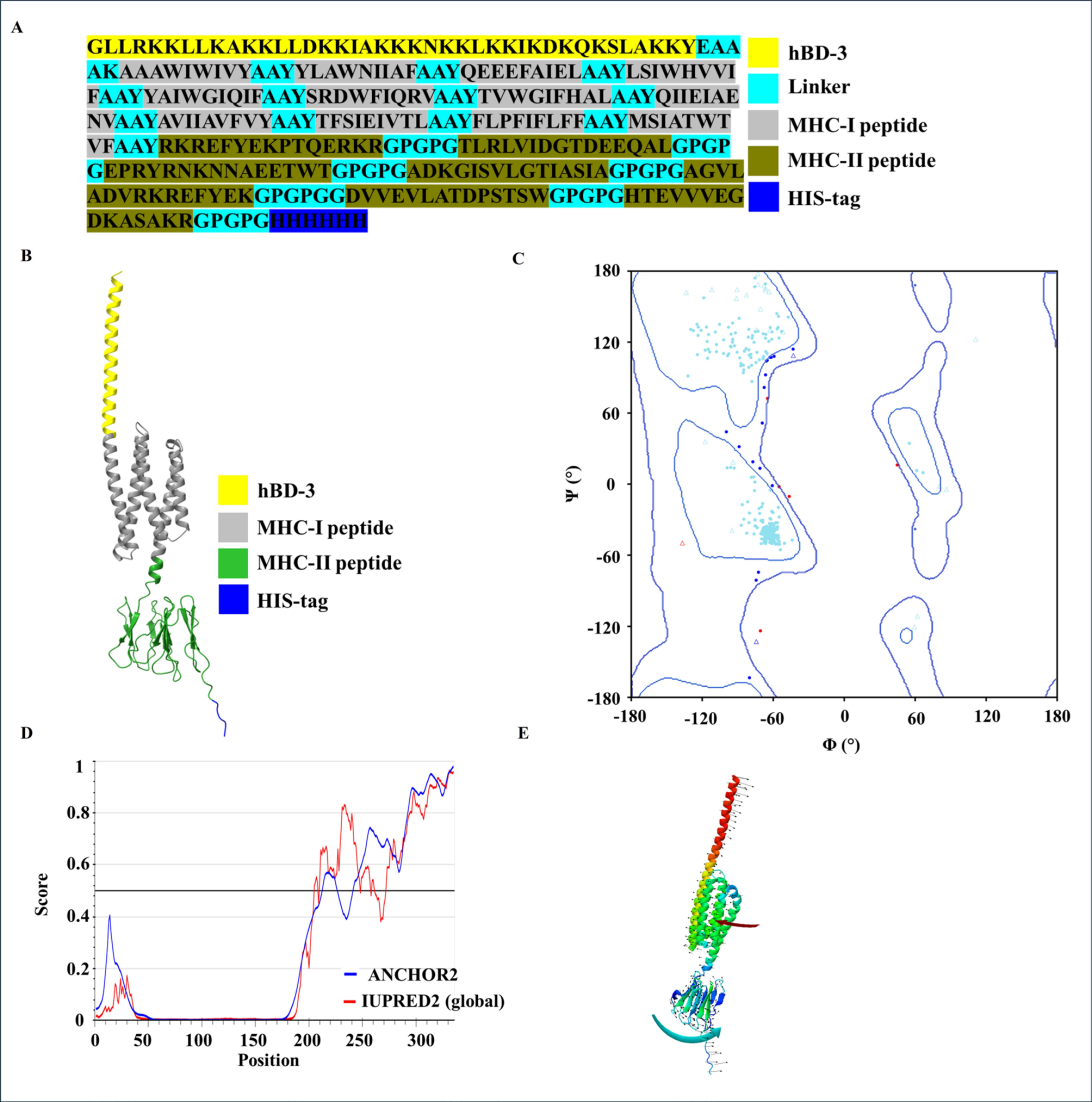
Structural and biophysical characterization of the proposed multi-epitope vaccine. (**a**) Primary sequence architecture. Schematic representation of the vaccine’s amino acid sequence. CTL and HTL epitopes are linked using appropriate flexible linkers, with human β-defensin 3 (hBD-3) incorporated at the N-terminus as an adjuvant and a C-terminal His tag added for purification. (**b**) Predicted tertiary structure. AlphaFold-generated 3D model of the vaccine construct. The hBD-3 adjuvant domain is shown in yellow, CTL-derived epitopes in grey, HTL-derived epitopes in green, and the C-terminal His tag in blue, illustrating the overall spatial organization. (**c**) Ramachandran plot evaluation. Ramachandran plot depicting the stereochemical quality of the predicted structure. Light blue points indicate residues in favored regions, dark blue points represent residues in allowed regions, and red points correspond to residues in disallowed regions. (**d**) Intrinsic disorder prediction. Intrinsically disordered regions were assessed using Anchor2 (blue) and IUPRED2 (red). Residues 0–200 show well-defined structural features with low disorder propensity, whereas residues 201–335 exhibit a high degree of predicted disorder, consistent with the AlphaFold model. (**e**) Normal mode analysis (NMA). Collective and local motions of the modeled protein were evaluated using the iMODS server. The structure illustrates the maximum displacement in the dominant normal mode. Global motions are highlighted with red and blue arrows, with finer local motions shown in black. Mobility ranges from low (blue) to moderate (green/yellow) to high (red).

The predicted 12 CTL epitopes, 7 HTL epitopes, and human beta defensin 3 (hBD3) were assembled into a multiepitope vaccine construct. The 12 CTL epitopes were linked together using the proteasomal cleavage linker AAY, while the 7 HTL epitopes were connected via the proteasomal cleavage linker GPGPG, which also maintains conformational flexibility. The hBD3 adjuvant was added to the N-terminal region to enhance immune activation, and a 6-His tag was appended to the C-terminal region for purification and identification purposes (Fig. 4a). The final assembled vaccine consisted of 335 amino acids.

The vaccine’s three-dimensional structure was predicted using AlphaFold-2. The resulting model displayed two distinct domains corresponding to the MHC-I and MHC-II peptides, which formed alpha helices and beta sheets interspersed with some disordered regions (Fig. 4b). Ramachandran plot analysis of the predicted 3D structure of MEV protein indicated that 93.49% of amino acid residues resided in the most favored regions (Fig. 4c, S4a, S4b), while additional allowed and disallowed regions accounted for 5.71% and 1.80% of residues, respectively. Intrinsically disordered region (IDR) analysis, performed using ANCHOR2 and IUPRED2 algorithms, was consistent with the AlphaFold prediction, revealing a highly ordered N-terminal region and a disordered C-terminal region (Fig. 4d). Normal mode analysis (NMA) of the assembled vaccine structure via the iMODS server indicated high structural mobility (Fig. 4e, S4c). The dominant functional flexibility was limited to a few large-scale movements, while the internal structure remained largely stable with only minor local fluctuations, as evidenced by the increased eigenvalues in higher modes (6–10) (Fig. S4c, S4d). Elastic network model analysis further supported the presence of flexible regions within the protein (Fig. S3d). Additionally, covariance matrix analysis revealed anti-correlated motions, with the two domains exhibiting opposite directions of movement (Fig. 4e, S4e). Taken together, these analyses suggest that the predicted structure is stable overall but contains flexible domains capable of dynamic movement.

### Physiochemical properties of the assembled multiepitope vaccine

The assembled multiepitope vaccine a calculated molecular weight of 37.01 kDa. The theoretical isoelectric point (pI) was determined to be 9.41, indicating the protein is basic. The amino acid composition analysis revealed a predominance of alanine (15.5%), glycine (9.0%), lysine (8.7%), isoleucine (8.1%), and leucine (6.3%). Positively charged residues (arginine and lysine) were present at a total of 42, while negatively charged residues (aspartic acid and glutamic acid) totaled 31, contributing to the overall positive charge of the protein. The protein’s atomic composition yields a molecular formula of C₁₇₃₄H₂₆₃₂N₄₄₄O₄₅₅S₁ with a total of 5266 atoms. The extinction coefficient at 280 nm was calculated as 84,800 M⁻^1^ cm⁻^1^, indicating significant absorbance useful for concentration determination in solution. The estimated half-life of the vaccine protein was predicted to be 30 h in mammalian reticulocytes (in vitro), over 20 h in yeast (in vivo), and over 10 h in Escherichia coli (in vivo), suggesting reasonable stability across different biological systems. The instability index was computed as 28.59, classifying the protein as stable. The aliphatic index was 89.58, indicating a potentially thermostable protein due to a high volume of aliphatic side chains. The grand average of hydropathicity (GRAVY) was -0.054, suggesting that the protein is slightly hydrophilic. Further analysis by ToxinPred, VaxiJen v2.0, and AllerTOP v2.0 tools predicted the vaccine to be non-toxic, probably antigenic, and non-allergenic. Protein solubility analysis using SolPro predicted that the protein is likely to be soluble upon overexpression, with a solubility probability of 0.72.

### Molecular dynamics (MD) simulation of multiepitope vaccine

**Fig. 5:**
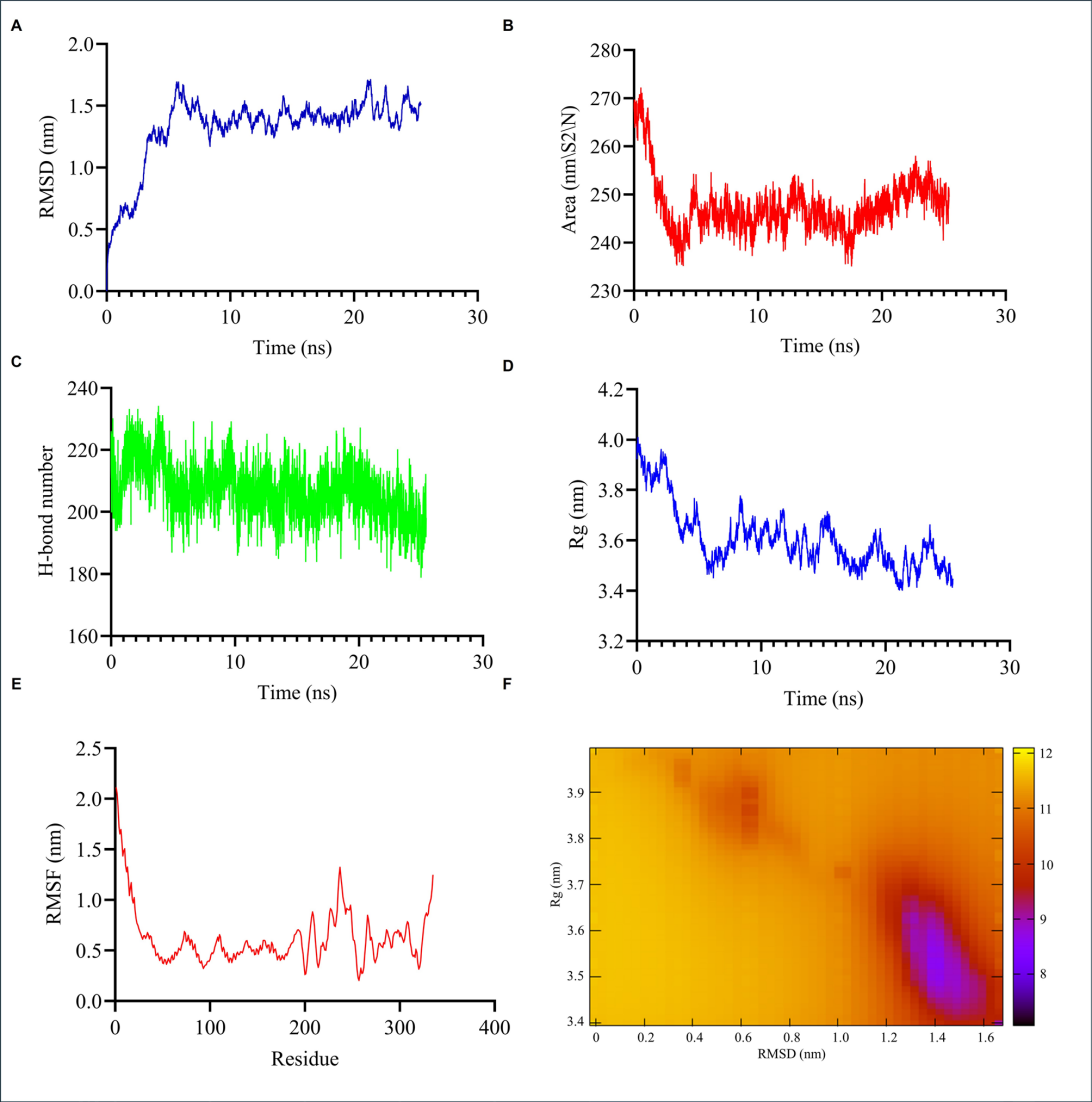
Molecular dynamics simulation analyses of the predicted MEV protein. (**a**) Root mean square deviation (RMSD). RMSD plot showing the structural stability of the MEV protein throughout the simulation. The system reaches equilibration at approximately 5 ns, after which fluctuations remain minimal, indicating stable conformational behavior. (**b**) Solvent-accessible surface area (SASA). SASA analysis demonstrates consistent solvent exposure of the MEV protein. The SASA values stabilize after ~ 5 ns, supporting the establishment of a stable structural conformation. (**c**) Hydrogen bond dynamics. Time-resolved plot of the number of hydrogen bonds formed by the MEV protein throughout the simulation. The relatively stable hydrogen bond count indicates persistent intra-molecular interactions and supports the overall structural stability of the protein. (**d**) Radius of gyration (Rg). Rg profile of the MEV protein over a 25-ns trajectory. The Rg values stabilize after approximately 5 ns, indicating sustained protein compactness and supporting the overall structural stability observed during the simulation. (**e**) Root mean square fluctuation (RMSF). RMSF analysis illustrates residue-level flexibility across the simulation. Regions with lower RMSF values correspond to rigid structural elements, while higher values indicate flexible or disordered regions. (**f**) Free energy landscape (FEL). FEL plot depicting the conformational space sampled by the MEV protein, mapped as RMSD versus Rg. Blue regions represent energetically favorable and highly stable conformations, orange indicates intermediate-energy states, and red regions reflect less stable, metastable conformations.

The multiepitope vaccine protein reached structural equilibrium at approximately 5 ns, as indicated by the plateau in the RMSD curve. The RMSD increased from 0.4 nm at the start of the simulation (0 ns) to approximately 1.4 nm at 5 ns, after which it remained stable for the remainder of the 25 ns MD simulation (Fig. 5a). The solvent-accessible surface area (SASA) decreased from approximately 270 nm^2^ to 245 nm^2^ and remained relatively stable, fluctuating around 245 nm^2^ for the remainder of the simulation (Fig. 5b). Together, the RMSD and SASA results suggest that the multiepitope vaccine protein quickly reaches a stable conformation early in the molecular dynamics simulation and maintains that stability over time. The number of intramolecular hydrogen bonds remained relatively stable, fluctuating between 200 and 220 throughout the simulation (Fig. 5c). The radius of gyration (Rg) decreased from 4.0 nm to approximately 3.5 nm and remained consistent for the remainder of the simulation, indicating a more compact structure (Fig. 5d). Similarly, the root mean square fluctuation (RMSF) dropped from 2.0 nm to approximately 0.5 nm and stayed stable, reflecting reduced flexibility and enhanced structural stability of the vaccine protein (Fig. 5e). The free energy landscape (FEL) plotted using RMSD and Rg revealed a prominent energy minimum (blue region) centered around RMSD = 1.4 nm and Rg = 3.5 nm, indicating the most thermodynamically stable conformation of the vaccine construct (Fig. 5f). This minimum corresponds with the plateaued RMSD and stable Rg observed during the MD simulation, supporting the conclusion that the vaccine protein adopts a compact and stable structure.

### Principal component analysis reveals dominant motion patterns of the vaccine construct

**Fig. 6:**
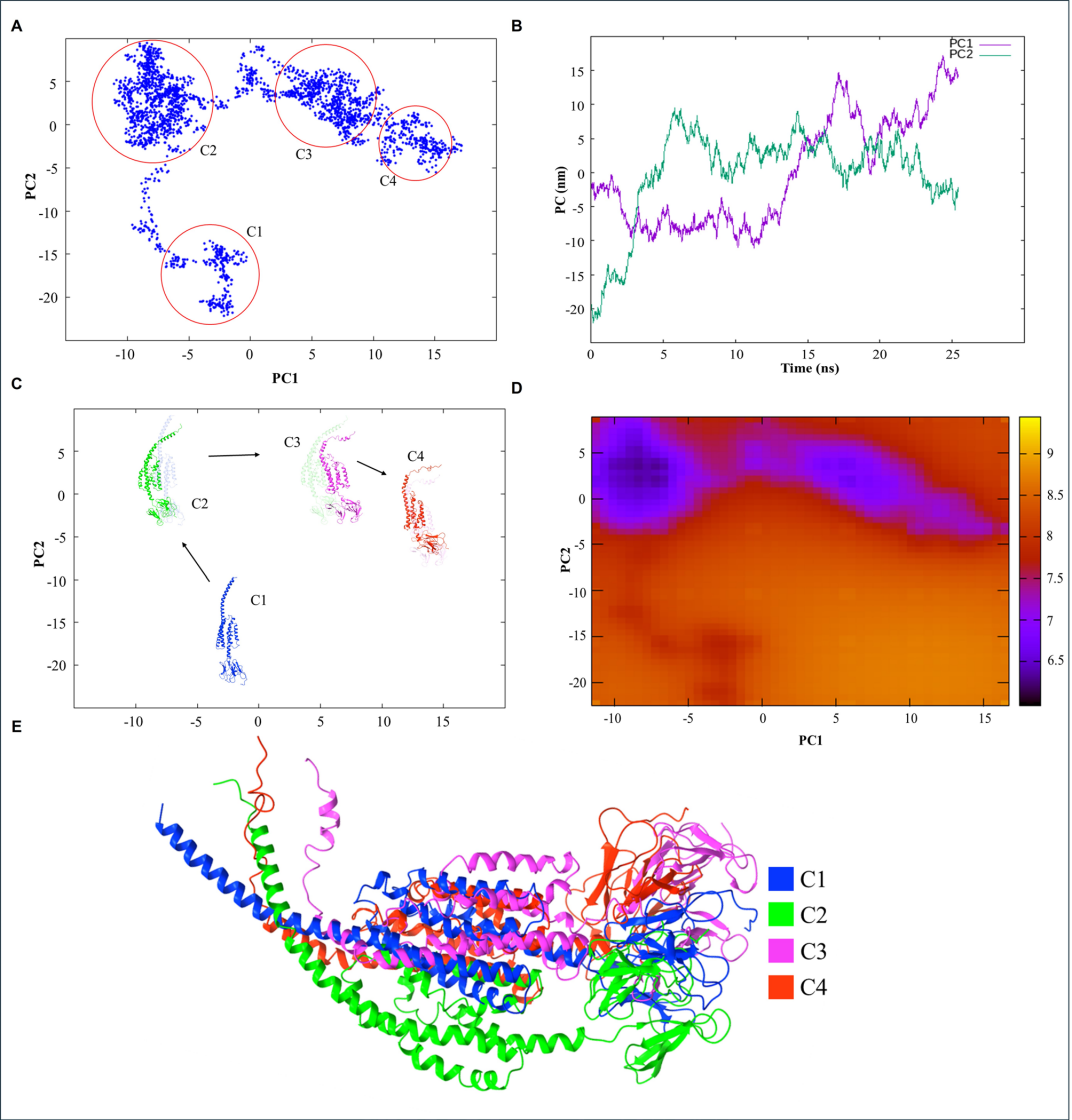
Principal component analysis (PCA) of MEV protein during simulation. (**a**) PCA clustering of conformational states. Principal component analysis of the simulation trajectory reveals four distinct conformational clusters, representing the major stable states sampled by the MEV protein. (**b**) Temporal evolution of principal motions. Time-resolved variation of the first two principal components, PC1 (purple) and PC2 (green), illustrating the dominant large-scale motions of the protein over the course of the simulation. (**c**) Representative structures of each cluster. Average structures derived from each PCA-defined cluster are shown in blue (cluster 1), green (cluster 2), pink (cluster 3), and red (cluster 4), highlighting the characteristic conformations associated with each state. (**d**) Free energy landscape (FEL) based on PCA. FEL plotted as PC1 versus PC2. Blue regions correspond to low free-energy basins (stable conformations), orange regions represent intermediate-energy states, and red regions denote higher-energy, less stable or metastable conformations. Cluster 2 occupies the most stable free-energy basin. (**e**) Structural overlay across clusters. Superimposed average structures from all four clusters demonstrate minimal overall structural deviation, supporting the conclusion that the MEV protein maintains consistent conformational stability throughout the simulation.

Principal component analysis (PCA) of the multiepitope vaccine protein revealed four distinct conformational clusters in the PC1 vs. PC2 plot (Fig. 6a). The temporal evolution of PC1 and PC2 was further analyzed to assess large-scale conformational motions during the simulation. PC1 remained relatively stable until approximately 10 ns, after which it gradually increased, indicating a slow conformational drift. In contrast, PC2 stabilized earlier, fluctuating between 0 and 5 after 5 ns, suggesting limited motion along this axis (Fig. 6b). Using the PCA data from Fig. 6b, four stable conformations were extracted from the MD simulation (Fig. 6c). Overall, the protein maintained structural stability throughout the simulation, with only minor movements predominantly observed in the N-terminal region. The free energy landscape (FEL) constructed using PC1 and PC2 revealed distinct low-energy basins corresponding to the four conformational clusters identified in the PCA (Fig. 6d). These regions are highlighted in blue, indicating thermodynamically favorable and stable states of the vaccine protein. The presence of well-defined blue basins suggests that the protein predominantly occupies these stable conformations during the simulation. Among these, cluster 2 exhibited the darkest blue region, indicating it represents the most thermodynamically stable conformation of the vaccine protein. The other clusters also corresponded to low-energy states but with relatively higher free energy. This pattern suggests that the protein predominantly occupies cluster 2 during the simulation, with occasional transitions to other metastable conformations. To compare the structural differences among the four stable conformations identified by PCA and FEL analysis, the representative structures were superimposed (Fig. 6e). The overlay revealed a high degree of structural similarity overall, with the most noticeable variations occurring in the N-terminal region, consistent with the observed flexibility in this area during the simulation.

### Immune simulation

**Fig. 7:**
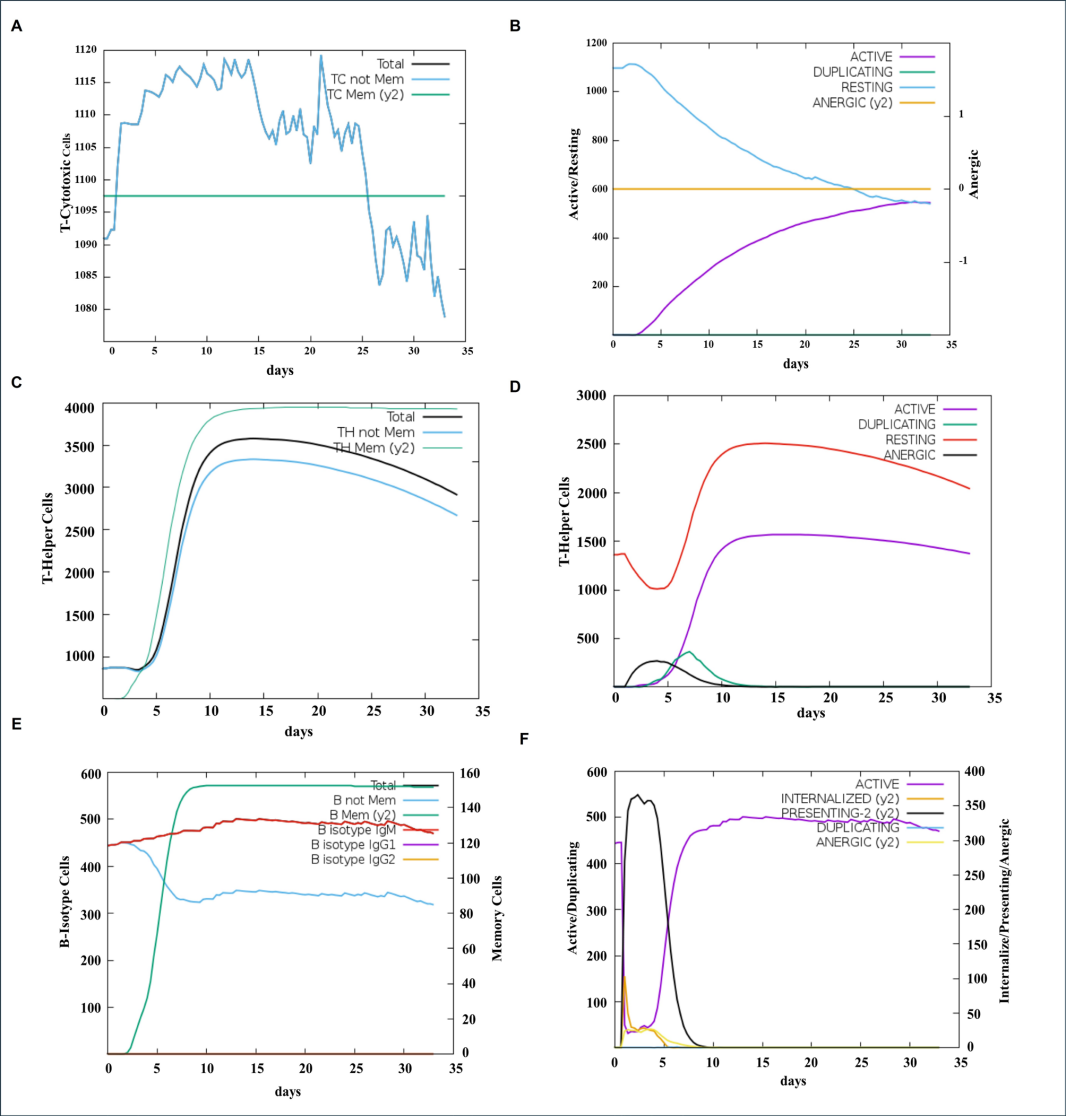
Simulated immune response to the multi-epitope vaccine using C-ImmSim. (**a**) Cytotoxic T lymphocyte (CTL) response. Following antigen injections, a sharp increase in non-memory CTLs (blue, left) is observed, accompanied by elevated levels of active CTLs (purple, left), indicating a strong and rapid cellular immune response. (**b**) Helper T lymphocyte (HTL) response. Both non-memory (blue, left) and memory (green, left) HTLs expand after antigen administration. The population includes active HTLs (purple, right) and resting HTLs (red, right), reflecting a balanced and sustained helper T cell-mediated response. (**c**) B cell response. Memory B cells (green, left) increase significantly following antigen exposure. Active B cells (purple, right) and antigen-presenting B cells (black, right), particularly those presenting Y2 epitopes, also rise markedly, indicating robust humoral immune activation and effective antigen presentation.

The immune simulation using C-ImmSim demonstrated a coordinated humoral and cellular immune response following administration of the multiepitope vaccine. Antigen levels declined rapidly and were completely cleared by day 5 post-injection, accompanied by a sharp rise in IgM levels, indicating a strong primary humoral response (Fig. S5a). This early IgM surge reflects effective antigen recognition and processing by the immune system. Notably, the cytokines IFN-γ and IL-2 exhibited the most prominent increases, reflecting strong cellular immune activation and support for T cell proliferation (Fig. S5b). Other cytokines, including IL-23, IL-18, IFN-β, IL-6, TNF-α, and IL-4, showed no significant change, while IL-12, TGF-β, and IL-10 displayed modest increases, indicating a balanced immune response. This profile suggests a Th1-biased immune response with coordinated cellular and humoral activation, without major cytokine imbalances (Fig. S5b). Cytotoxic T lymphocyte (CTL) populations increased significantly, with active CTLs rising sharply within 2 days post-vaccination as the resting CTL population declined (Fig. 7a). This early expansion suggests a rapid and robust cytotoxic response. Additionally, T helper cells expanded around day 5, including both active and resting subsets (Fig. 7b, left). Memory T helper cells also proliferated notably between days 5 and 10, indicating sustained immune activation and memory formation (Fig. 7b, right). Similarly, B memory cells increased, supporting the establishment of long-term humoral immunity (Fig. 7c, left). B cell numbers rose following vaccination, along with a sharp increase in antigen-presenting activity that peaked early and declined by day 10, consistent with efficient immune priming (Fig. 7c, right). Overall, the simulation graphs capture immune responses at early, intermediate, and late phases post-vaccination, showing coordinated activation of adaptive immunity while natural killer (NK) cell and macrophage populations remained stable (Fig. S5c-d). These results collectively indicate that the vaccine effectively stimulates adaptive immune components, including antibody production, T cell activation, memory cell development, and antigen presentation, while maintaining a stable innate immune baseline.

## Discussion

Globally, infectious diseases remain the second leading cause of death. *A. baumannii*, a Gram-negative coccobacillus, thrives under aerobic conditions. Over the past decade, significant research advancements have deepened our understanding of *A. baumannii*, providing detailed insights into its pathogenesis^15^. This pathogen poses a serious global health threat due to its unique ability to rapidly acquire multidrug resistance, including resistance to critical antibiotics such as carbapenems, colistin, and tigecycline^16^. Because of these characteristics, *A. baumannii* has emerged as a formidable pathogen and is currently listed on the World Health Organization’s Priority Pathogen List for vaccine development. Vaccine development presents a promising strategy to combat this growing threat. Vector and component-based vaccines offer more targeted effects compared to live attenuated vaccines. Previous research has effectively developed vaccine designs against a variety of pathogens, including *Neisseria gonorrhoeae* and other therapeutically relevant bacteria and viruses, using reverse vaccinology and multi-epitope prediction pipelines^17–21^. For instance, recent research has shown that immunological simulation, structural modeling, and epitope screening are useful for logical vaccine design^17^. Conserved vaccine targets and multi-epitope vaccines with high anticipated immunogenicity and population coverage have been developed using similar computational techniques^17,18^. Further research has demonstrated the value of combining antigenicity, allergenicity, and docking analyses for candidate selection^19,20^, and confirmed the usefulness of immunoinformatics workflows for viral vaccine development^21^.

However, approaches that focus on a single protein often result in only partial protection against infection. Previous vaccine studies against *A. baumannii* have primarily focused on outer membrane vesicles, whole-cell antigens, or a limited number of outer membrane proteins, which provide only narrow or limited protective efficacy and coverage^22–24^. Therefore, there is a critical need for an innovative vaccine capable of targeting multiple strains of multidrug-resistant *A. baumannii*. Recent advances in vaccine research have emphasized MEVs, which use recombinant proteins or peptide fragments, offering a safer and more effective alternative. To develop MEVs targeting *A. baumannii*, we employed a novel in silico approach, focusing on upregulated genes associated with antibiotic resistance.

In this study, we systematically screened genes upregulated during carbapenem resistance to identify non-homologous, antigenic proteins as potential vaccine candidates against *A. baumannii*. This approach integrates antibiotic resistance dynamics with immunogenic potential, increasing the likelihood of targeting proteins that are both functionally important during infection and accessible to immune recognition. Notably, unlike most previous MEV studies that focused primarily on surface proteins, our strategy targets meropenem-induced, non-homologous proteins that are conserved across multiple virulent strains. By directly linking resistance phenotype with immunogenic targets, our method enhances the relevance of candidate antigens and addresses the challenge of strain variability in vaccine design.

While upregulated gene expression often correlates with elevated protein abundance, this relationship is not always direct due to factors such as post-transcriptional regulation and protein degradation. Ideally, designing vaccine constructs based on proteomic data would provide a more accurate reflection of antigen availability during infection^25–27^. Nonetheless, transcriptomic profiling remains a valuable and widely accepted substitution for identifying potential targets, particularly when proteomic datasets are limited or unavailable^28^. Future studies incorporating proteomic validation will be essential to refine and confirm the expression of candidate proteins under antibiotic stress, thereby enhancing the accuracy and effectiveness of vaccine design.

To minimize the risk of cross-reactivity, we carefully screened and excluded proteins with significant sequence similarity to human and gut microbiome proteins during antigen selection. This approach enhances the specificity and safety of the vaccine candidates by minimizing unintended host or commensal reactivity. Conservation analysis across multiple virulent *A. baumannii* strains supports the broad applicability of the selected candidates, helping to overcome the key challenge of strain heterogeneity in vaccine design. We combined CTL and HTL epitope prediction with molecular docking, MD simulation, and immune simulation to develop a rational, multi-layered strategy maximizing immune coverage and efficacy.

The integrated in silico results demonstrated a stable vaccine construct with strong MHC interactions and robust adaptive immune responses. The immune simulation indicated prominent IFN-γ and IL-2 induction, alongside modest increases in IL-12, TGF-β, and IL-10, while IL-23, IL-18, IFN-β, IL-6, TNF-α, and IL-4 showed no significant changes. This cytokine profile suggests a Th1-biased response with coordinated cellular and humoral activation, without major cytokine imbalances. CTL and helper T cell populations expanded robustly, and B memory cells increased, supporting long-term adaptive immunity. NK and macrophage populations remained stable, reflecting a balanced innate immune baseline. The innate immune compartment includes NK cells and macrophages, which may not be significantly stimulated in silico unless the vaccine construct specifically includes pathogen-associated molecular patterns (PAMPs) or innate adjuvants. Since our construct is composed of selected peptide epitopes linked with human β-defensin-3 (an adaptive adjuvant), innate immune activation was not strongly represented.

Additionally, the use of predictive algorithms for epitope binding and toxicity, while robust, is limited by the inherent constraints of current computational models and the variability of human HLA alleles. In this study, we utilized multiple common HLA alleles from both class I and class II loci to enhance the breadth of epitope binding predictions. However, given the extensive polymorphism of HLA genes across different populations, expanding the allele coverage further will be important to ensure broader vaccine applicability.

Our structural evaluations via molecular docking, AlphaFold modeling, and molecular dynamics simulation further support the structural integrity of the vaccine construct. The compact, energetically favorable conformation observed during MD simulations, along with stable RMSD, Rg, and SASA profiles, indicates that the vaccine rapidly adopts a thermodynamically stable form. Principal component analysis revealed limited motion confined largely to the N-terminal region, while the free energy landscape identified discrete low-energy basins corresponding to stable conformational states. These results collectively suggest that the vaccine is not only structurally stable but also exhibits favorable flexibility for efficient antigen processing and presentation.

We utilized exclusively in silico methods to design a potential multiepitope vaccine (MEV) against *A. baumannii* (Fig S6). Similar computational approaches have been applied to pathogens such as *Streptococcus gallolyticus*^29^, *Mycoplasma pneumoniae*^30^, *Klebsiella aerogenes*^31^, *Schistosoma* species^32^, and *Campylobacter jejuni*^33^. While bioinformatics significantly accelerates vaccine development by narrowing down candidates and forecasting immune responses, it is important to emphasize that this work remains entirely computational. The predictions and models generated here provide a foundational framework but require extensive validation through in vitro and in vivo experiments to confirm immunogenicity, safety, and protective efficacy^34,35^. Animal models will be essential to evaluate the biological relevance of the vaccine construct, including its capacity to produce robust cellular and humoral immune responses against *A. baumannii* infection. Without such empirical validation, the clinical applicability of the vaccine remains hypothetical.

Moreover, vaccine development extends far beyond candidate identification, requiring iterative optimization, preclinical testing, clinical trials, and regulatory approvals all of which are time-consuming and costly. Nevertheless, the application of computational approaches, such as those demonstrated in this study, helps shorten the early phases of vaccine design by providing rapid, cost-effective insights into antigen selection, epitope mapping, and structural stability. This rational, data-driven strategy ultimately contributes to more focused and efficient downstream experimental work, accelerating the path toward a viable vaccine candidate. Furthermore, the computational pipeline developed here is adaptable to other antibiotic-resistant pathogens on the WHO priority list, making it a versatile tool for rational vaccine discovery. This study was the first step of developing a novel MEV against *A. baumannii* and further validation of the targets is essential for its biological relevance. In vivo infection models will be helpful to evaluate the surface accessibility and immunogenicity, whereas quantitative proteomics and Western blotting can confirm protein expression and stability. These experimental methods will offer important proof of the MEV’s potential for protection.

In conclusion, our study presents a novel and rational approach to identify vaccine targets linked to antibiotic resistance mechanisms in *A. baumannii*. By prioritizing proteins upregulated during meropenem exposure and validating their immunogenic potential through multi-epitope design and simulation, we provide promising vaccine candidates that may help address the urgent global threat posed by multidrug-resistant *A. baumannii*. The insights gained here lay the groundwork for future empirical testing and development of effective vaccines to combat multidrug-resistant *A. baumannii*.

## Materials & methods

### RNA-Seq data processing and differential gene expression analysis

RNA-seq data corresponding to 9-h treatments, with and without meropenem, were downloaded from the GEO database under accession number GSE190441. Raw sequencing reads were aligned to the *A. baumannii* ATCC 17,978 reference genome (RefSeq accession ASM1337208v1) using Bowtie2 (v2.5.1)^36^ with default parameters. Alignment quality was assessed using Samtools (v1.17). Gene-level read counts were quantified using featureCounts^37^ from the Subread package (v2.0.3). Only uniquely mapped reads were included; multi-mapping reads were excluded from downstream analyses. Differential gene expression analysis was performed using the DESeq2 package (v1.40.2) in R^38^. Gene annotations, including protein names and product descriptions, were extracted from the ASM1337208v1 GFF3 file. Data visualizations were generated using ggplot2 (v3.4.4) and EnhancedVolcano (v1.27.0). Gene expression levels (FPKM with standard deviation error) were plotted using GraphPad Prism 9.

### Pathway enrichment analysis

Differentially expressed genes (DEGs) with an adjusted p-value < 0.05 and |log₂ fold change|≥ 1 were annotated with KEGG Orthology (KO) identifiers. KO terms corresponding to upregulated and downregulated genes were analyzed separately using the clusterProfiler package (v4.8.1) in R^39^. Pathway enrichment was performed using the enrichKEGG function to enable mapping against general bacterial KEGG pathways. Significantly enriched pathways were identified based on Benjamini–Hochberg adjusted p-values < 0.05. Enrichment results for upregulated and downregulated gene sets were visualized using dot plots and bar plots created with ggplot2.

### Homology filtering

Protein sequences corresponding to significantly upregulated genes were retrieved from the NCBI protein database and compiled in FASTA format. To assess potential homology with the human host and gut microbiota, BLASTp (v2.13.0) analyses were performed against two custom protein databases: the human proteome derived from the GRCh38 (hg38) reference genome, and the Unified Human Gastrointestinal Protein (UHGP-90) database. Proteins with ≥ 30% sequence identity and ≥ 50% alignment coverage to any human protein were classified as potentially cross-reactive and excluded. Proteins below both thresholds were designated as non-homologous (“safe”) and retained for further consideration as therapeutic or vaccine candidates.

### Antigenicity and allergenicity prediction

Antigenicity and allergenicity of the non-homologous selected protein sequences were evaluated using VaxiJen v2.0^40^ and AllerTOP v2.0^41^, respectively. Antigenicity was assessed using the alignment-independent VaxiJen v2.0 server (http://www.ddg-pharmfac.net/vaxijen/VaxiJen/VaxiJen.html). The bacterial model was selected, with a threshold of 0.5 to identify probable antigens. Allergenicity was predicted using AllerTOP v2.0 (https://www.ddg-pharmfac.net/AllerTOP/). Proteins predicted as non-allergens were selected for further analysis.

### Protein conservation among virulent strains

The non-allergen proteins were further analyzed for conservation across different virulent strains of *A. baumannii*, including AB30, AB5075, ACICU, AYE, D1279779, HUMC1, and LAC4. Conservation was assessed using BLASTp by aligning the selected protein sequences against the proteomes of each strain. Sequence identity values were visualized using a heatmap generated with the ggplot2 package in R.

### Phylogenetic analysis

Molecular Evolutionary Genetic Analysis Version X (MEGA X) software (http://www.megasoftware.net) was used for the Phylogenetic analysis of various *A. baumannii*’s strains to access their genetic diversity^42,43^. Briefly, reference sequence for each strain was retrieved from the NCBI GenBank, aligned by ClustalW algorithm in MEGA and Maximum Likelihood tree was constructed with 1000 bootstraps having defaults value^44,45^. Next, we constructed a phylogenetic tree of 23 protein candidates for *A. baumannii* using NGPhylogeny.fr (https://ngphylogeny.fr/) with default options and was annotated using iTOL (Interactive Tree Of Life) version 5^46,47^.

### Cytotoxic T cell epitope prediction

Cytotoxic T cell epitope prediction was performed using the NetMHCpan tool (https://services.healthtech.dtu.dk/service.php?NetMHCpan-4.1)^48^. The analysis included the following human HLA class I alleles: HLA-A01:01, HLA-A02:01, HLA-A03:01, HLA-A24:02, HLA-A26:01, HLA-B07:02, HLA-A*11:01, HLA-B08:01, HLA-B27:05, HLA-B15:01, HLA-B58:01, HLA-B40:01, and HLA-B39:01. Peptides of nine amino acids in length were selected for analysis. Predicted epitopes were subsequently evaluated for immunogenicity using the IEDB Immunogenicity (http://tools.iedb.org/immunogenicity/)^49^ and for toxicity using ToxinPred ((https://crdd.osdd.net/raghava/toxinpred/)^50^ .

### Helper T cell epitope prediction

Helper T cell epitope prediction was carried out using NetMHCIIpan (https://services.healthtech.dtu.dk/service.php?NetMHCIIpan-4.0) ^51^ against a set of common HLA class II alleles: DRB101:01, DRB101:03, DRB104:01, DRB107:01, DRB108:01, DRB109:01, DRB110:01, DRB111:04, DRB112:01, DRB113:01, DRB114:01, DRB115:01, DRB301:01, DRB401:01, and DRB5*01:01. Peptides of 15 amino acids in length were selected for analysis. Immunogenicity and toxicity analyses were performed as described in the Cytotoxic T Cell Epitope Prediction section.

### Epitope population coverage prediction

Population coverage of the selected epitopes was assessed using the IEDB Population Coverage tool (IEDB, Population coverage; https://www.iedb.org/), with allele frequencies obtained from the Allele Frequency Net Database. The combined class I and class II epitope allele mappings were used to calculate projected global coverage (%), average epitope HLA hits per individual, and pc90 values.

### Epitope structure prediction

The three-dimensional structures of predicted T-cell epitopes were modeled using the PEP-FOLD3.5 server (https://mobyle.rpbs.univ-paris-diderot.fr/cgi-bin/portal.py#forms::PEP-FOLD3)^52^ with default parameters. The best-ranked models, based on sOPEP energy scores, were selected for downstream docking analysis.

### MHC molecule preparation and molecular docking

Crystal structures of human major histocompatibility complex class I and II molecules were obtained from the Protein Data Bank (PDB IDs: 1AKJ for MHC class I and 1DLH for MHC class II). The peptide-binding grooves were isolated by removing co-crystallized ligands and extraneous chains, retaining only the α- and β—domains that form the peptide-binding cleft using ChimeraX (v1.9). Molecular docking of the modeled epitopes into the MHC binding grooves was performed using the HADDOCK 2.4 web server^53^. Protein–peptide complexes were prepared in PDB format and submitted with active and passive residues defined around the binding cleft. The standard docking protocol was followed, consisting of rigid-body docking, semi-flexible refinement, and final refinement in water. Top docking clusters were ranked based on HADDOCK scores. Docking results were quantitatively analyzed based on the HADDOCK score, van der Waals energy, buried surface area (BSA), and root-mean-square deviation (RMSD) of the clusters. These metrics were extracted from the HADDOCK output and visualized using the ggplot2 package in R.

### Assembly of the multi-epitope vaccine and structure determination

A multi-epitope vaccine construct was designed to include cytotoxic T lymphocyte (CTL) epitopes, helper T lymphocyte (HTL) epitopes, an immunostimulatory adjuvant, and a purification tag. Human Beta-Defensin 3 (hBD-3) was incorporated at the N-terminus as an adjuvant to enhance innate immune responses. CTL epitopes were linked using an AAY linker to promote proteasomal processing and efficient MHC class I presentation, while HTL epitopes were joined using a GPGPG linker. For purification and identification purposes, a 6xHis-tag was added at the C-terminus of the construct. The three-dimensional structure of the vaccine was predicted using AlphaFold2^54^ to assess the overall folding and spatial arrangement of epitopes. Structural validation was performed using a Ramachandran plot to evaluate the stereochemical quality. Intrinsically disordered regions within the construct were identified using the IUPRED3 server (https://iupred3.elte.hu/)^55^, aiding in the prediction of flexible or unstructured segments. Additionally, structural refinement and flexibility analysis were conducted using the iMODS server (https://imods.iqf.csic.es/)^56^ to evaluate the dynamic behavior and stability of the vaccine model. Physicochemical characteristics and overall stability of the vaccine were analyzed using the ProtParam tool (https://web.expasy.org/protparam/)^57^. To confirm the solubility of the construct, the SOLpro server (http://scratch.proteomics.ics.uci.edu/)^58^ was further employed.

### Molecular dynamics simulation

Molecular dynamics (MD) simulations were carried out using GROMACS version 2021.4^59^. The MEV protein structure was modeled and simulated using the CHARMM36 force field. The system was solvated in a cubic box of TIP3P water molecules, maintaining a minimum distance of 1.0 nm between the solute and the box edges. Na⁺ and Cl⁻ ions were added to neutralize the system and mimic physiological conditions. Energy minimization was performed using the steepest descent algorithm until the maximum force on the system was below 1000 kJ/mol/nm. Equilibration was carried out in two phases: 100 ps under constant volume (NVT) and 100 ps under constant pressure (NPT), both at 310 K and with position restraints applied to the heavy atoms of the protein. The temperature and pressure were maintained using the V-rescale thermostat and Parrinello–Rahman barostat, respectively.

The 25 ns production MD run used a leap-frog integrator with a 2 fs timestep. All bonds involving hydrogen atoms were constrained using the LINCS algorithm (order 4, one iteration). Electrostatic interactions were computed using the Particle Mesh Ewald (PME) method with a real-space cutoff of 1.0 nm. Lennard–Jones interactions were truncated at 1.0 nm, with analytical dispersion corrections applied to energy and pressure. Periodic boundary conditions were applied in all directions, and the Verlet cutoff scheme was used for neighbor searching.

### Trajectory analysis

Post-simulation analysis was conducted using GROMACS built-in tools. Structural stability was evaluated using root mean square deviation (RMSD), root mean square fluctuation (RMSF), radius of gyration (Rg), and solvent-accessible surface area (SASA). Hydrogen bond dynamics were analyzed over the trajectory using geometric criteria. To explore the conformational landscape, free energy landscapes (FEL) were constructed as functions of RMSD vs. Rg and principal components (PC1 vs. PC2) obtained via principal component analysis (PCA). Clustering analysis was performed based on the major principal components to identify dominant conformational states during the simulation. All plots were generated using GROMACS or GraphPad Prism 9.

### Immune simulation

The immune response was simulated using the C-ImmSim server^60–63^ with a random seed of 12,345, a simulation volume of 10, and 100-time steps. The host HLA profile was set to include MHC class I alleles HLA-A01:01, HLA-A02:01, HLA-B07:02, and HLA-B08:01, along with MHC class II alleles HLA-DRB101:01 and HLA-DRB103:01. A single injection of vaccine (without LPS) was administered at time step 2, consisting of 1000 antigen units and an adjuvant concentration of 100. Immune cell dynamics, cytokine levels, and antibody responses were monitored over the simulated period.

## Supplementary Information

Supplementary Information.

## Data Availability

All data generated in the current study is available in the manuscript or can be obtained from the authors on request.
