# Supplementary material for: In silico design of a multiepitope vaccine against antibiotic drug-resistant Acinetobacter baumannii
Source: Sci Rep. 2026 Apr 3;16:14151. doi: 10.1038/s41598-025-30795-8 (PMC13139457; doi:10.1038/s41598-025-30795-8)
Supplement: Supplementary file 1 — Supplementary Information. [file 41598_2025_30795_MOESM1_ESM.docx]

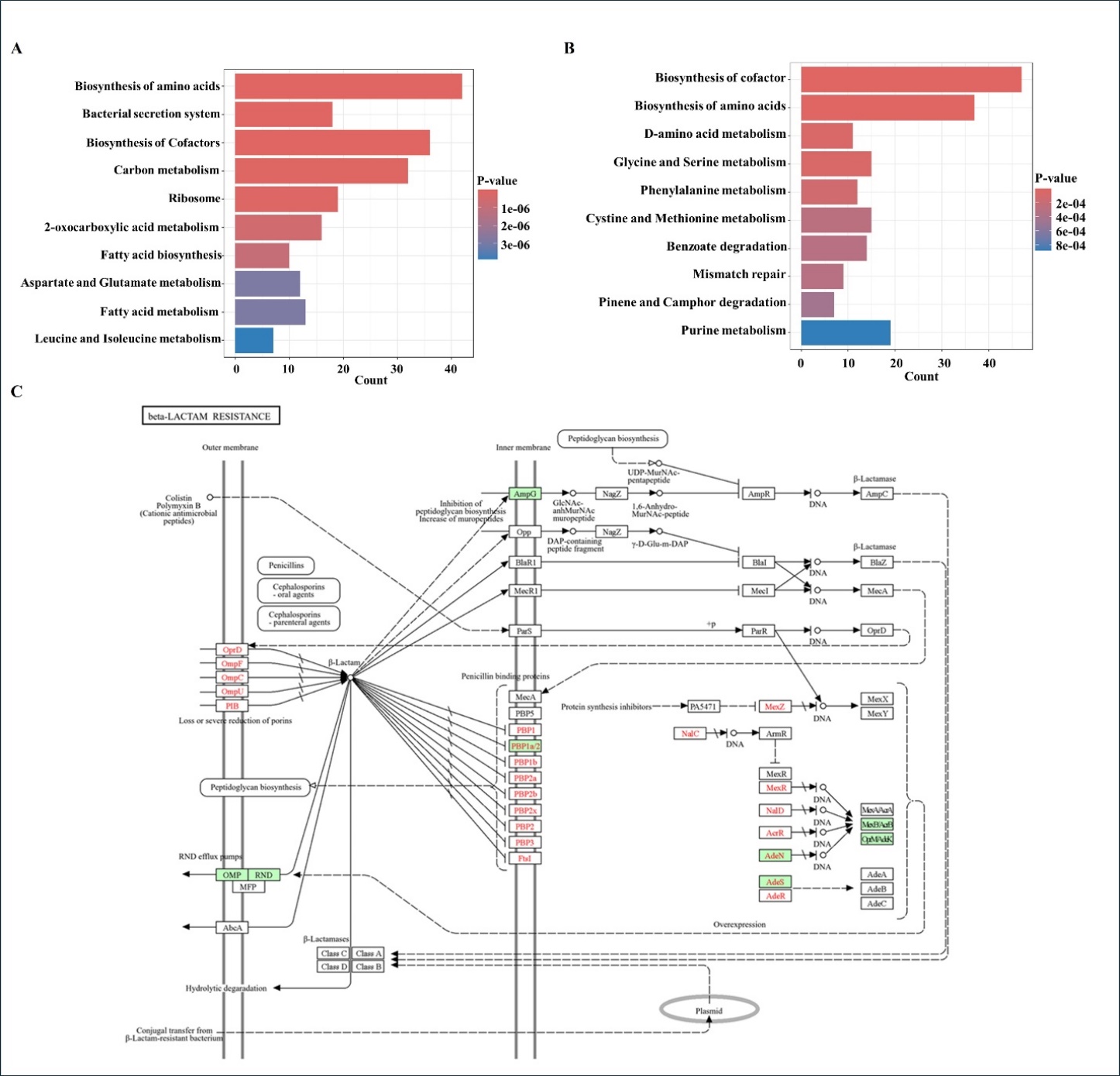


**Figure S1. Transcriptomic and functional analyses of *A. baumannii* following meropenem exposure.**

(a) GO enrichment of upregulated genes. Gene Ontology (GO) enrichment analysis of genes significantly upregulated after meropenem treatment.

(b) GO enrichment of downregulated genes. GO enrichment analysis of significantly downregulated genes.

(c) KEGG pathway annotation of carbapenem resistance. BLAST KOALA-based KEGG annotation of the carbapenem resistance pathway illustrates upregulation of outer membrane proteins (OMPs) and resistance-nodulation-cell division (RND) efflux pump components. KEGG pathway database permission was granted with reference number 252969 [64,65].


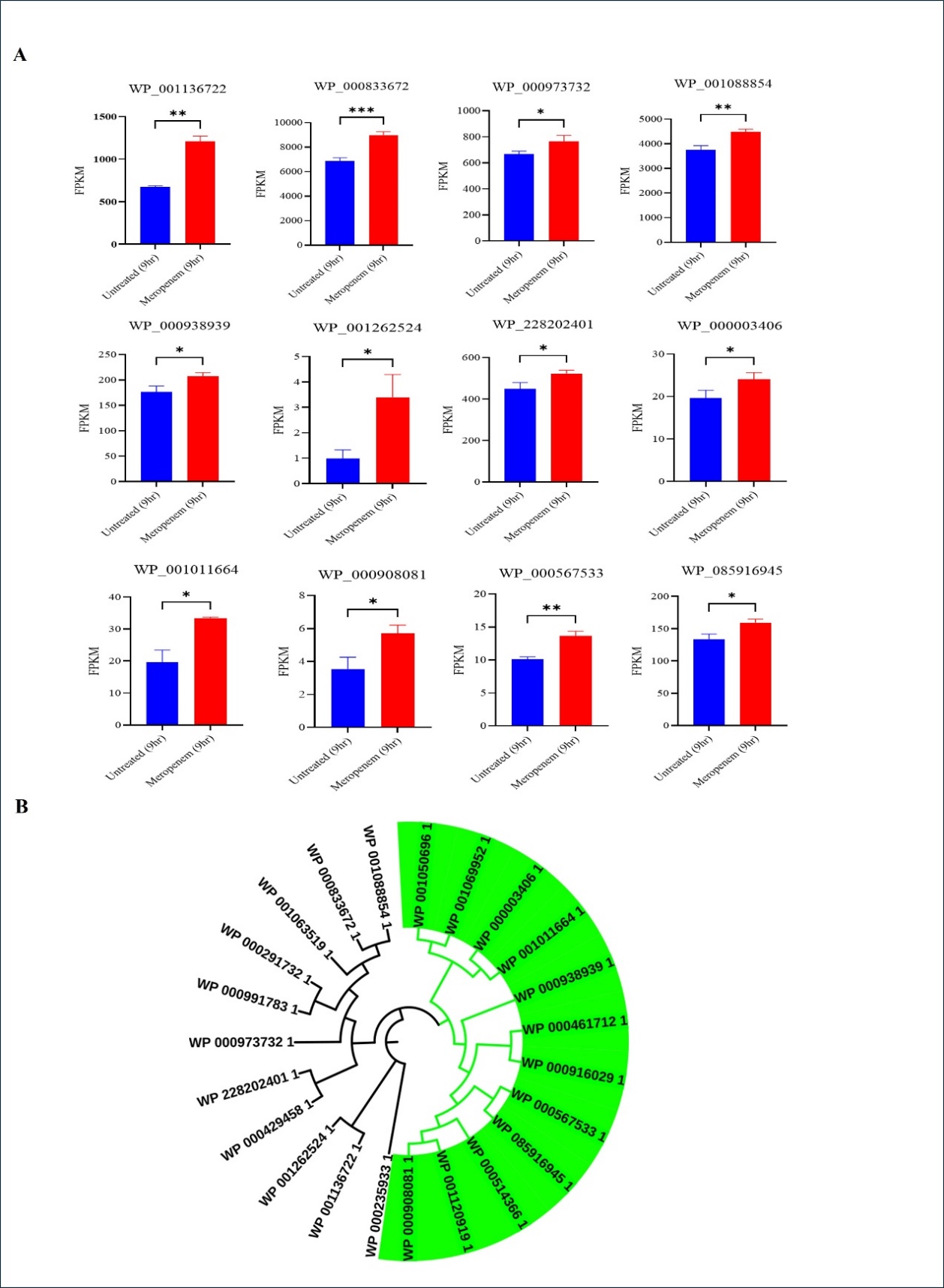


**Figure S2. Expression and phylogenetic analysis of candidate vaccine antigens in *Acinetobacter baumannii*.**

(a) Expression profiling of candidate genes. Normalized expression levels (FPKM) of 12 overexpressed candidate genes are shown, with protein names indicated for clarity. Statistical significance was determined using an unpaired t-test, highlighting genes with elevated transcription after meropenem exposure.

(b) Phylogenetic relationships of vaccine candidates. Phylogenetic tree of 23 non-allergenic protein candidates selected for vaccine development. The tree was constructed using NGPhylogeny.fr and annotated with iTOL version 5, illustrating evolutionary relationships among the candidate antigens.


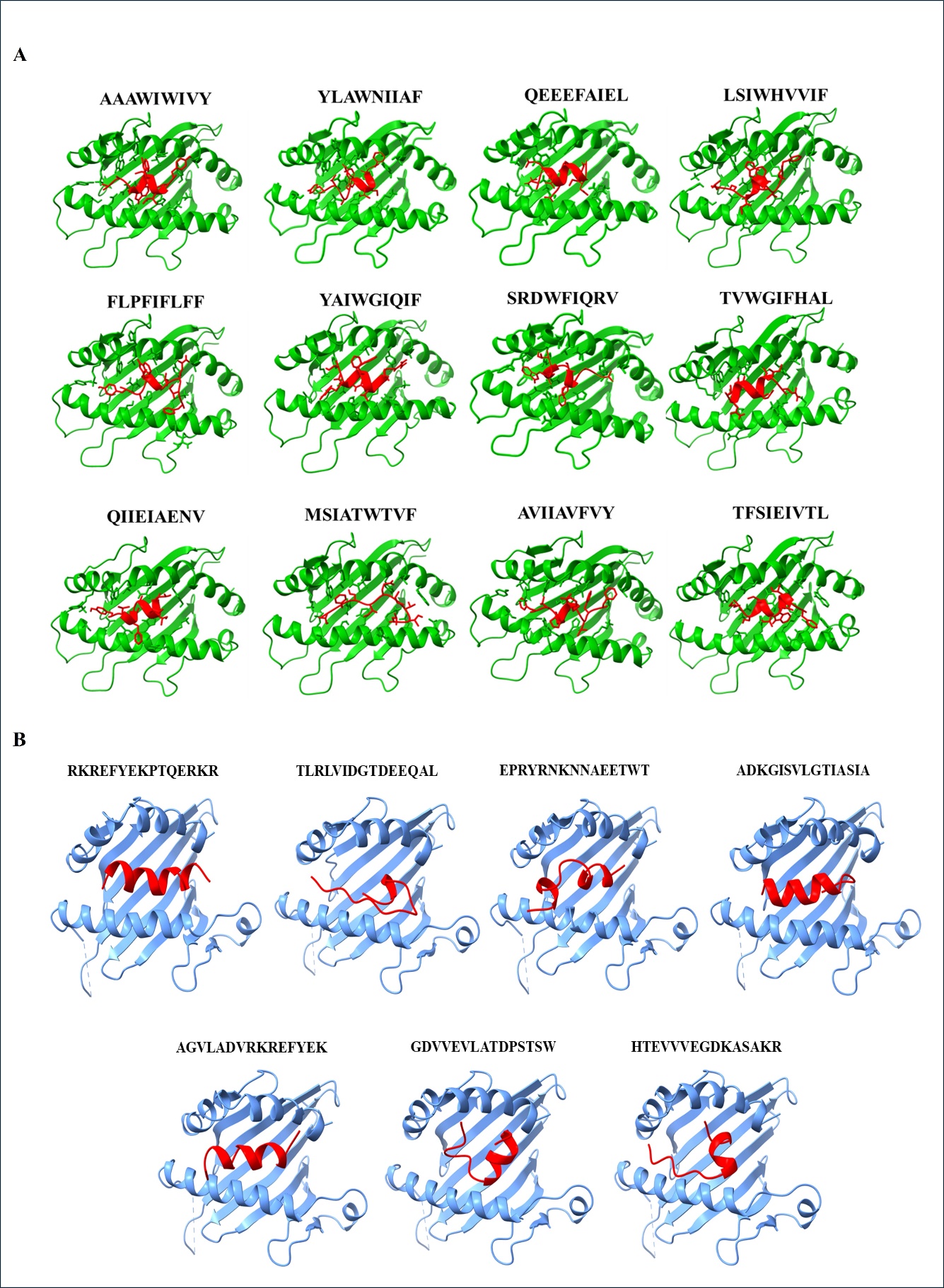


**Figure S3. Representative molecular docking of T-cell epitopes with MHC molecules.**

(a) CTL peptide–MHC class I docking. Representative HADDOCK docking model showing the binding orientation of the top-scoring cytotoxic T lymphocyte (CTL) peptide (red) within the MHC class I binding groove (green), highlighting key positioning and interactions.

(b) HTL peptide–MHC class II docking. Representative HADDOCK docking model illustrating the binding of the top-scoring helper T lymphocyte (HTL) peptide (red) within the MHC class II binding groove (cornflower blue), emphasizing favorable docking orientation and potential stabilizing contacts.


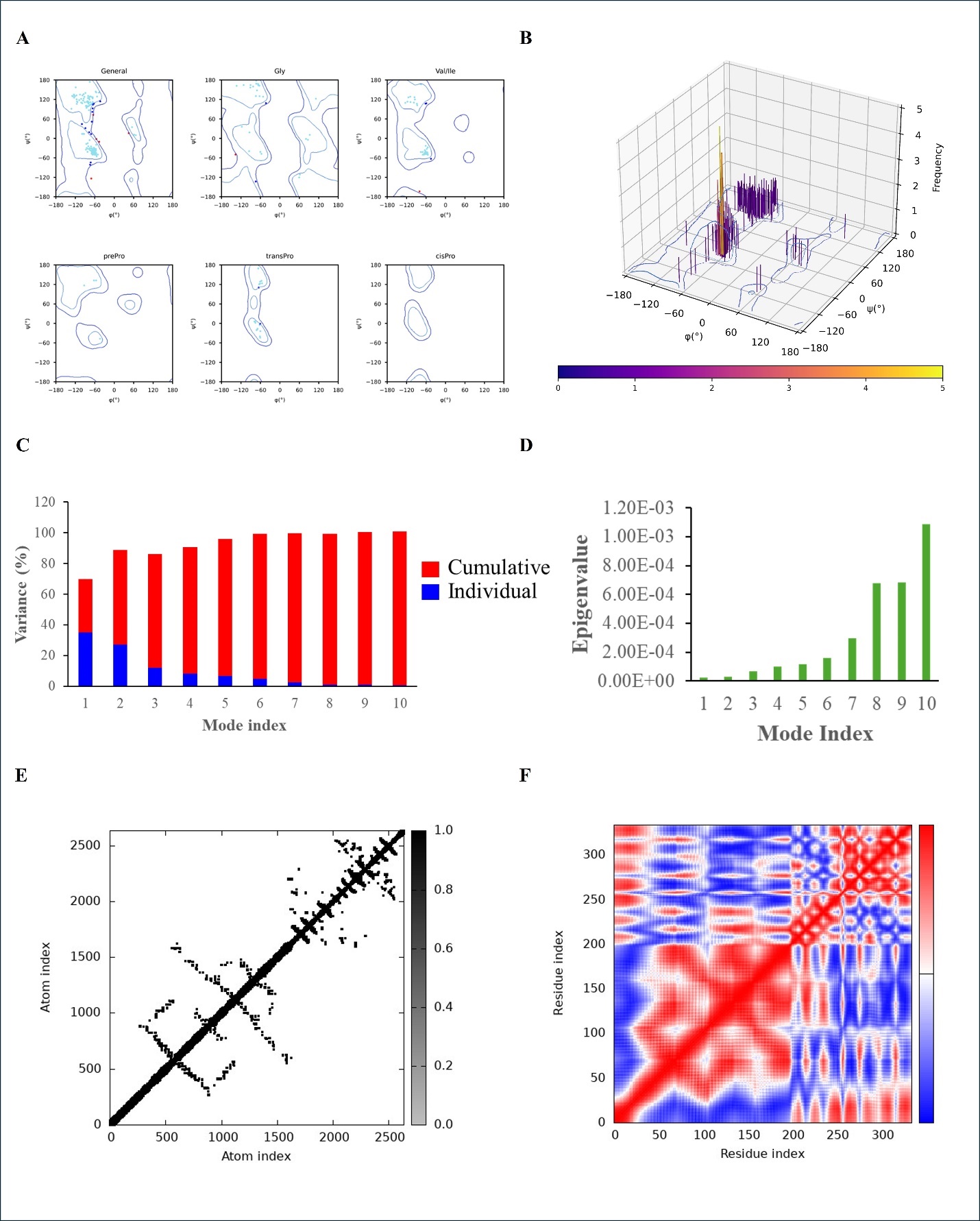


**Figure S4. Structural validation and dynamic analysis of the predicted vaccine construct.**

(a) Ramachandran plot of the predicted structure. Plot of the AlphaFold-modeled vaccine structure. Light blue dots indicate residues in favored regions, dark blue dots represent allowed regions, and red dots correspond to disallowed regions for general amino acids, glycine, valine/isoleucine, pre-proline, trans-proline, and cis-proline residues.

(b) 3D Ramachandran plot. Three-dimensional representation focusing on general amino acid residues, providing a detailed view of backbone dihedral angle distributions.

(c) Normal mode variance. Variance analysis from iMODS showing individual mode contributions (blue) and cumulative variance, illustrating the distribution of motion across the normal modes.

(d) Eigenvalue analysis. Eigenvalues from iMODS reflect the stiffness of protein motion in each normal mode. Lower eigenvalues correspond to larger-amplitude, easier motions, whereas higher eigenvalues indicate stiffer, more constrained movements.

(e) Elastic network model. Representation of the predicted vaccine structure as a spring-based network, where Cα atoms (dots) act as nodes, modeling residue-level flexibility and connectivity.

(f) Convergence/correlation matrix. iMODS-derived matrix showing correlations between residue pairs. Blue indicates highly correlated motions (similar directional movement), while red indicates low or uncorrelated motions, reflecting independent or opposing residue movements.


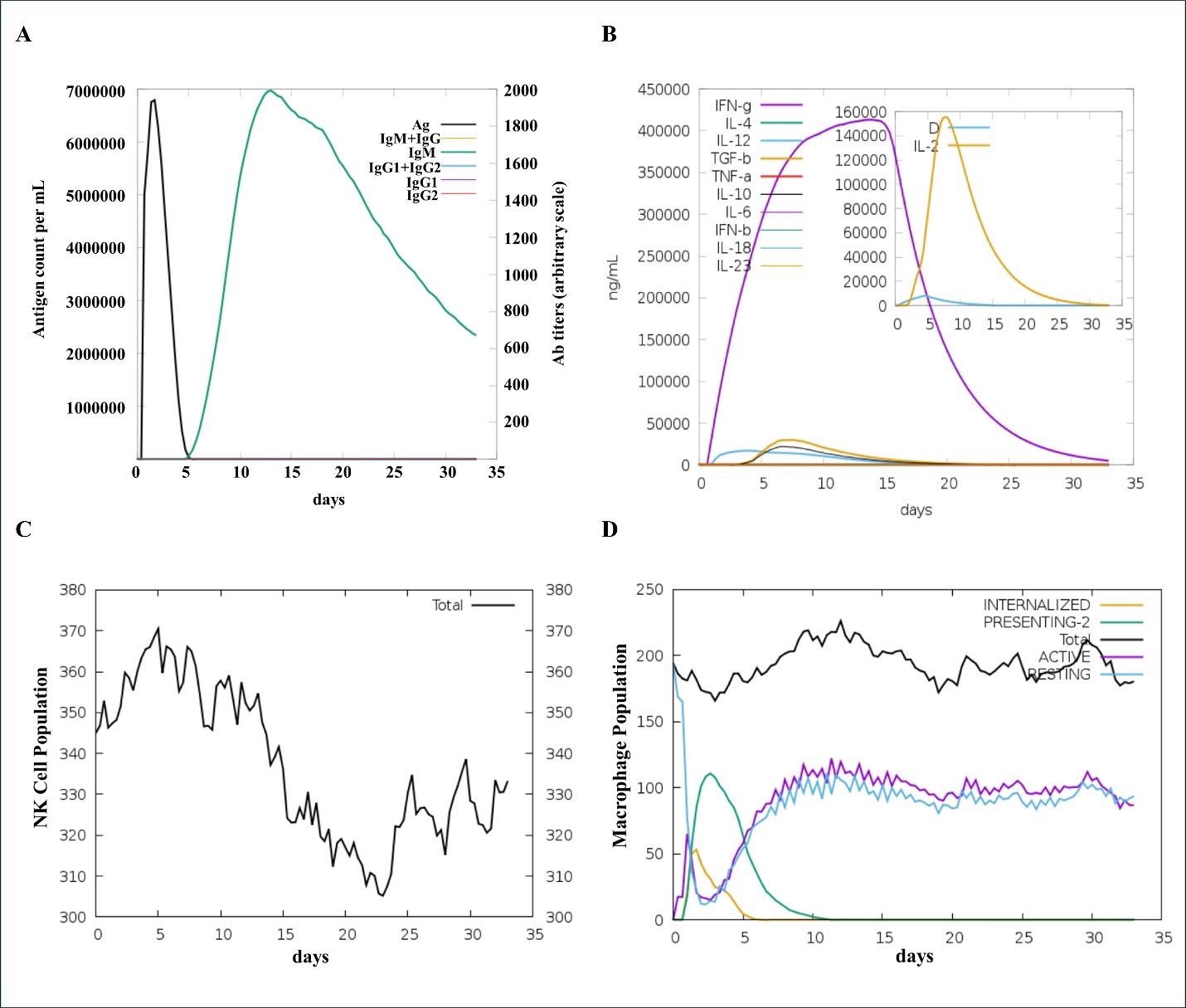


**Figure S5. Humoral, cytokine, and innate immune responses predicted by C-ImmSim simulation.**

(a) Antigen and immunoglobulin response. Simulated antigen (Ag, black) and immunoglobulin (IgM, green) levels following vaccine administration. Antigen concentration declines rapidly after each injection, indicating prompt immune recognition and clearance, while IgM levels rise after antigen clearance, reflecting an effective early humoral response.

(b) Cytokine profile. Levels of IFN-γ (purple) and IL-2 (yellow) increase following antigen exposure, suggesting activation of cellular immunity and support for T cell proliferation.

(c) Natural killer (NK) cell response. NK cell levels (black) remain largely unchanged throughout the simulation, indicating minimal involvement in the modeled immune response.

(d) Macrophage response. Macrophage levels remain stable during the simulation, reflecting a consistent innate immune background without significant activation.


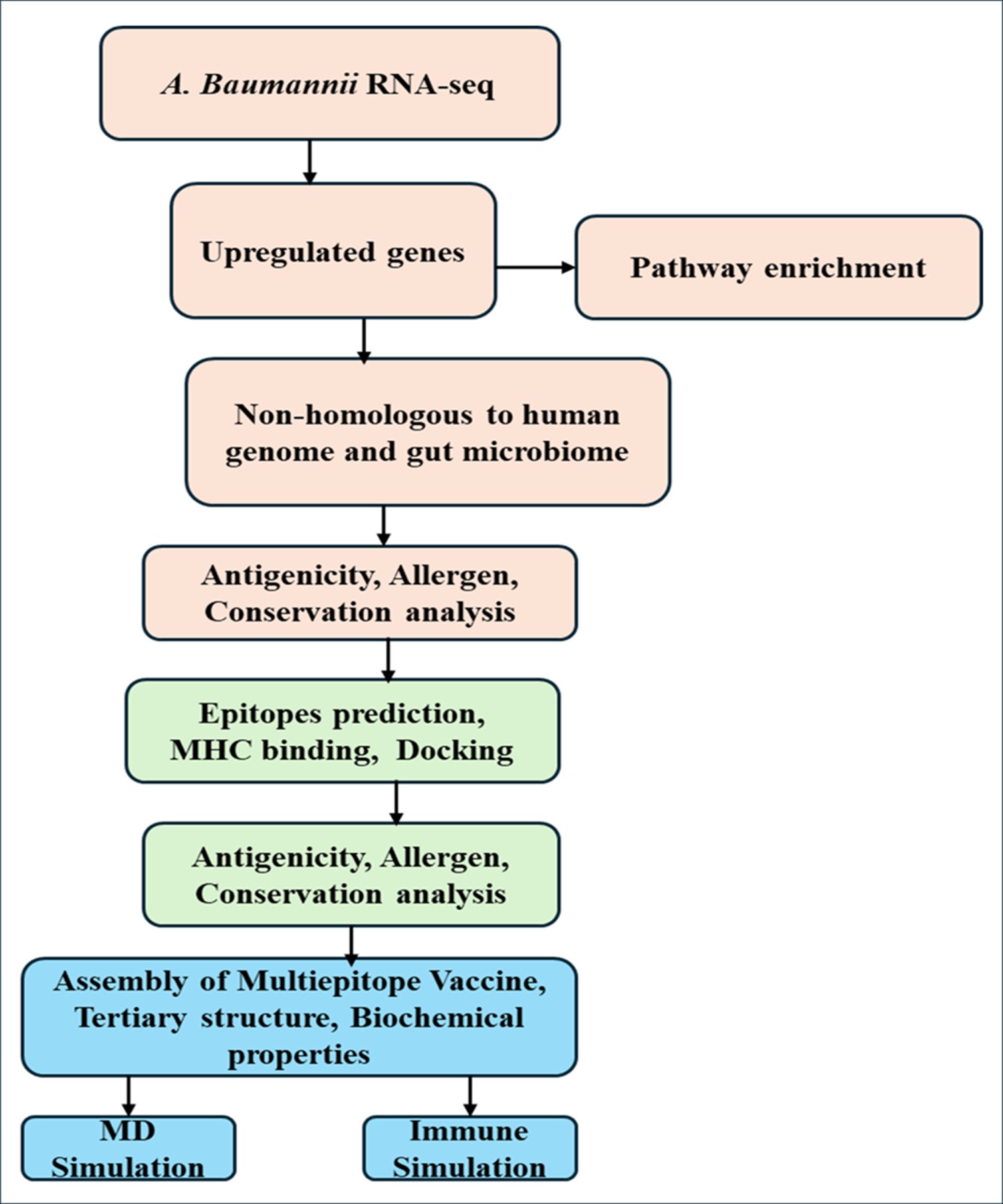


**Figure S6. Schematic representation of the vaccine construct.**
